# Revealing hidden protonated conformational states in RNA dynamic ensembles

**DOI:** 10.1093/nar/gkaf1366

**Published:** 2025-12-18

**Authors:** Ainan Geng, Rohit Roy, Laura Ganser, Linshu Li, Hashim M Al-Hashimi

**Affiliations:** Department of Biochemistry, Duke University School of Medicine, Durham, NC 27710, United States; Center for Genomic and Computational Biology, Duke University School of Medicine, Durham, NC 27710, United States; Department of Biochemistry, Duke University School of Medicine, Durham, NC 27710, United States; Department of Biochemistry and Molecular Biophysics, Columbia University, New York, NY 10032, United States; Department of Biochemistry and Molecular Biophysics, Columbia University, New York, NY 10032, United States

## Abstract

Identifying protonated states within RNA ensembles, quantifying their p*K*_a_s, and elucidating the kinetic mechanisms by which they form is essential for understanding protonation-coupled biochemical reactions and how RNAs sense and adapt to pH fluctuations. However, detecting protonated states is challenging when they are short-lived and lowly populated. Here, using pH-dependent NMR chemical exchange, kinetic solvent isotope effects, and mutation, we show that a low-populated (0.4% at pH 6.4) conformational state of HIV-1 TAR RNA is coupled to protonation of a C⁺–C mismatch. Despite an intrinsic p*K*_a_ of ~7.1, the energetic penalty to form this alternative conformation depressed the apparent p*K*_a_ to ∼4.0, below the pH range typically probed experimentally. Substituting C–C with a G–C base pair abolished the pH-dependence of these dynamics, confirming C–C as the protonation site. This hidden protonated state competes with a more abundant conformation harboring a C–A⁺ mismatch, producing a non-monotonic ensemble response to pH. Both transitions follow an induced-fit mechanism, in which solvent-exposed nucleobases are rapidly protonated followed by slower changes in secondary structure. These findings reveal a general mechanism for protonation-coupled conformational switching in RNA and provide a framework for dissecting sparsely populated protonated states and their multi-protonation-state dynamics.

## Graphical Abstract

**Graphical Abstract:**
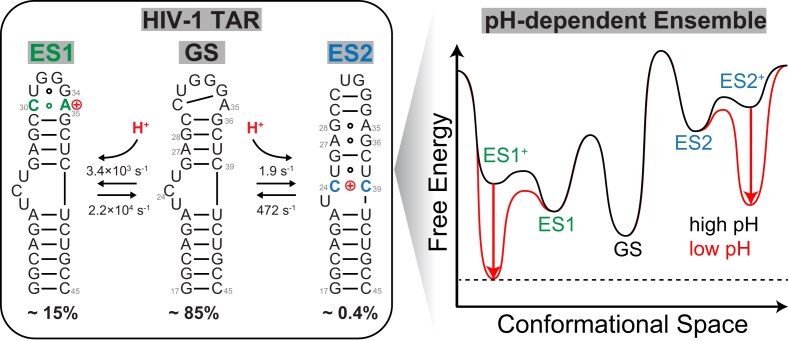

## Introduction

RNA molecules exist as dynamic ensembles of interconverting conformations, many of which are lowly populated and short-lived [1–7], yet play crucial roles in how RNAs fold [1–3] and interact with proteins and ligands [4–6] and are frequently the functional state responsible for driving biochemical processes [4, 8]. These alternative higher-energy conformations, referred to as excited conformational states (ESs) [9], frequently arise by replacing canonical Watson–Crick base pairs (bp) in the dominant ground state (GS) with non-canonical mismatches [1, 6, 7, 10]. Many of these mismatches, including G–A [1, 5, 7], C–A [1, 11–14], U–C [15], and C–C [10, 16, 17], can be stabilized by protonation of one of the nucleobases. As a result, the dynamics by which RNAs adopt these ESs are frequently coupled to nucleobase protonation and influenced by pH [1, 5, 7, 11, 18]. Identifying which states within RNA ensembles are protonated, pinpointing their protonation sites, quantifying their p*K*_a_s, and elucidating the kinetic mechanisms by which they form is essential not only for building a deep, predictive understanding of RNA ensemble behavior, but also for dissecting the mechanisms of protonation-coupled biochemical reactions and regulatory processes, and for harnessing these transitions in synthetic biology [19] and in the development of RNA-targeted and RNA-based therapeutics [1, 14, 20–22].

Pronation-coupled changes in RNA secondary structure regulate key biological processes, including microRNA maturation [5, 23], RNA editing [24], the assembly and function of ribonucleoprotein complexes [25], and viral RNA translation and replication [1, 14, 26]. Protonation and deprotonation of nucleobases are not only central to the chemistry of acid-base catalysis in ribozymes [27, 28], but they also often trigger catalytically essential conformational changes. For example, deprotonation-coupled motions in mismatches such as G–U enable them to adopt Watson–Crick-like conformations, which are proposed to contribute to transcriptional [29] and translational errors [30, 31], and CRISPR off-target editing [32].

Identifying low-abundance protonated conformational states within RNA ensembles is also critical for elucidating how RNAs sense and adapt to pH fluctuations, and for linking protonation-coupled structural dynamics to cellular regulation and environmental adaptation [5, 26, 33]. While most RNAs function in near-neutral to mildly alkaline environments (pH ∼7.2–7.4), numerous physiological contexts expose them to more acidic conditions, which can stabilize protonated conformations that are otherwise sparsely populated at physiological pH [34]. For example, RNAs destined for degradation are trafficked to lysosomes, acidic cellular compartments with pH ∼4.5–5.0 [35–37], as part of the cellular quality control machinery that clears damaged or unwanted RNAs [38–40]. RNA viruses such as influenza and coronaviruses, along with RNA-based vaccines and therapeutics, also enter cells through acidic endosomal pathways [41–43]. In an evolutionary context, models of the early RNA world propose that RNA first emerged under acidic conditions, where protonated mismatches such as C⁺–C could have enabled non-enzymatic template-directed replication by stabilizing self-pairing of quasi-complementary, cytosine-rich sequences [44], such as 5′-GCC-3′, which drive tRNA anticodon dimerization under acidic pH [45]. Protonation-coupled conformational changes involving G–A and C–A mismatches can help regulate pre-microRNA processing in response to changes in cellular environment [5, 46]. Protonation-induced conformational dynamics in U6 RNA can lead to potential differences in splicing upon encountering different cellular stimuli [47]. Finally, proton-coupled conformational changes also enable nucleic acids to serve as highly sensitive riboswitches, capable of detecting and responding to pH fluctuations for cellular regulation and adaptation [26, 33].

**Figure 1.**
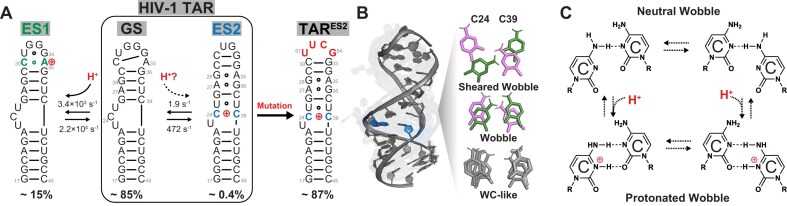
Proton-coupled secondary structural transitions in HIV-1 TAR RNA. (**A**) TAR exists in dynamic equilibrium between a dominant ground state (GS) and two excited conformational states with alternative secondary structures, ES1 and ES2. ES1 is coupled to formation of a protonated C30-A^+^35 mismatch in the apical loop. This work examines whether ES2 is also coupled to protonation of the C24–C39 mismatch. Also shown is that the TAR^ES2^ mutant predominantly forms the alternative ES2 conformation [10] through replacement of the UG ES2 apical loop with a thermodynamically more stable UUCG tetraloop, with the tetraloop residues labeled from 51 to 54. (**B**) Structural overlay of the N = 10 conformations in the FARFAR-MD-NMR ensemble of TAR^ES2^ [10], highlighting the conformations formed by the C24–C39 mismatch. Shown to the right are three representative geometries of the C–C mismatch, observed in the TAR^ES2^ ensemble, with varying amounts of relative shearing between the two nucleobases: sheared wobble with a shear of ∼5–7 Å, the wobble with a shear of ∼2 Å primed for protonation, and a Watson–Crick-like conformation with shear <1 Å. The wobble and sheared wobble conformations are colored pink or green to represent the two types of wobbles, where either the C24 (pink) or C39 (green) is sheared toward the major groove. (**C**) Chemical structures of the neutral and protonated C–C wobble conformations proposed to form in the protonated ES2.

Using NMR, we previously demonstrated that the HIV-1 transactivation response element (TAR) RNA [56, 59, 60] forms a transient conformational state referred to as “ES1”[1, 11], which exists at 15% population. ES1 forms by zipping up the hexanucleotide TAR apical loop via a protonated C30-A^+^35 mismatch (Fig. 1A), which has an intrinsic p*K*_a_ of ~7.5 and an apparent p*K*_a_ of 5.6 for the proton-coupled transition, which includes the contribution from conformational change. In addition to ES1, the TAR ensemble includes a second ES referred to as ES2 [10, 56, 60] (Fig. 1A). ES2 is significantly lower in population (∼0.4%)[10, 56, 60], and it forms by reshuffling bps throughout the bulge, upper stem, and apical loop, resulting in an alternative secondary structure enriched in non-canonical mismatches (Fig. 1A). While a functional role has yet to be assigned to either TAR ES1 or ES2, point substitution that renders ES2 the dominant state promotes kissing-loop dimerization [59] and potently inhibit cellular transactivation in a structure-dependent manner [61]. ES2 could potentially function as a switch modulating the binding affinity of TAR to the Tat-SEC complex, and is also of great interest for the design of anti-HIV therapeutics, which preferentially bind and stabilize this alternative inactive TAR conformation [61, 62].

NMR chemical exchange techniques are uniquely capable of detecting and characterizing these low-populated, short-lived, and protonated nucleic acid conformational states [25, 48–51]. These approaches exploit large changes in the NMR chemical shifts accompanying protonation of adenine-N1 when forming protonated G–A [1, 5, 7] and C–A [1, 11–13] mismatches. However, these NMR methods face challenges when the cytosine base is protonated, as occurs in C^+^–C and C^+^–U mismatches as well as G*_syn_*-C^+^ Hoogsteen bps, as this is associated with a smaller NMR chemical shift change for commonly measured nuclei [52–55]. The difficulty is compounded when the protonated state is exceptionally low in population, which can depress the apparent p*K*_a_ of the transition below the typical experimental pH range and make any pH dependence difficult to measure experimentally. Moreover, if this state coexists in dynamic equilibrium with more abundant protonated conformers, its signature may be masked or confounded in pH-dependent measurements [56, 57]. Even more challenging is dissecting the kinetic mechanisms by which protonated ESs form and determining whether protonation occurs before or after the conformational change [58]. This requires measuring kinetic rates involving fleeting intermediates, which have even lower populations and are shorter-lived, often differing from reactants by the mere addition of a single proton.

ES2 features three mismatches, which could in principle be protonated (Fig. 1A). While NMR chemical shift analysis previously ruled out protonated Hoogsteen conformations for the A27-G36 and G28-A35 mismatches [10], whether the C24–C39 mismatch was protonated remained unresolved. When embedded in Watson–Crick RNA and DNA duplexes under acidic pH conditions, the C–C mismatch can adopt a wobble conformation, similar to that observed for U–U mismatches [16, 52, 55, 63, 64], in which one of the cytosine-N3 nuclei is protonated with an apparent p*K*_a_ ranging between 6.7 [65] and 7.0 [55], allowing the formation of the second hydrogen bond.

The possibility that the C24–C39 mismatch might be protonated in TAR ES2 garnered further support from our recent determination of a conformational ensemble for a TAR mutant (TAR^ES2^), which stabilized ES2 as the dominant conformation (Fig. 1B) [10]. In this ensemble derived using the FARFAR-NMR-MD approach [10], the C24–C39 mismatch adopted the wobble geometry in ∼20% of the conformations, a pairing arrangement commonly associated with protonation of cytosine-N3 enabling formation of a second hydrogen bond (Fig. 1B). Given the unique challenges associated with detecting protonation in ES2, including its low population, dynamic competition with the more abundant protonated ES1⁺, and the weak NMR signature for protonated cytosine, this observation prompted us to revisit ES2 and directly investigate whether it is also coupled to protonation via the C24–C39 mismatch.

Using pH-dependent NMR chemical exchange, kinetic solvent isotope effects (KIE), and mutations that alter conformational equilibria, we demonstrate that TAR ES2 is also coupled to protonation of the C^+^24-C39 mismatch, which has an intrinsic p*K*_a_ ≥ 6.4, so that ≥50% of ES2 detected by NMR is protonated at pH = 6.4. Despite an intrinsic p*K*_a_ ≥ 6.4, the energetic penalty required to form ES2 depresses the overall p*K*_a_ for the protonation-coupled transition to ∼4.0, below the pH range typically probed experimentally. While direct experimental detection of this fleeting protonated C^+^–C mismatch proved difficult, given the comparatively small effect of protonation on commonly measured NMR chemical shifts, we were able to definitively verify the C–C mismatch as the protonation site by using a mutant substituting the C–C mismatch in ES2 with a neutral G-C pair, which abolished the pH-dependence of this conformational transition. The hidden protonated ES2 dynamically competes with the more abundant ES1 involving the protonated C–A^+^ mismatch, producing a complex, non-monotonic ensemble response to pH. As for ES1, for ES2 protonation also occurs via an induced-fit mechanism: the unpaired C24 bulge residue in the TAR GS is initially protonated, followed by a slower, rate-limiting conformational rearrangement that forms ES2. Our findings reveal a potentially general mechanism for protonation-coupled conformational switching in RNA and provide a framework for revealing sparsely populated protonated states and dissecting multi-state protonation-coupled dynamics.

## Materials and methods

### RNA Preparation

#### *In vitro* transcription

Uniformly ^13^C/^15^N-labeled TAR and RNAs, as well as AUG- and C-type labeled TAR, were synthesized by *in vitro* transcription using T7 RNA polymerase (New England BioLabs), synthetic DNA template (Integrated DNA Technologies) containing the T7 promoter sequence (TTAATACGACTCACTATA), and uniformly labeled (^13^C/^15^N) nucleotides (Cambridge Isotope Laboratories, Inc.). The DNA templates for the two fully labeled samples included a 2′-O-methyl modification on the last two 5′ terminal nucleotides to avoid non-templated dangling ends [66]. The transcription reactions were carried out at 37°C for 16 h. All RNAs were purified using a 20% (w/v) denaturing polyacrylamide gel with 8M urea and 1× TBE [Tris/borate/ethylenediaminetetraacetic acid (EDTA)]. The RNAs were extracted from the excised gel by electro-elution (Bio-Rad) followed by concentration and ethanol precipitation. The RNAs were then annealed in water at 95°C for 5 min and snap-cooled on ice for 1 h. Finally, RNAs were buffer exchanged using an Amicron Ultra-15 centrifugal filter into NMR buffer (15 mM sodium phosphate, 25 mM sodium chloride, 0.1 mM EDTA) with varying pH (5.4–8.4). 10% (v/v) D_2_O was added to each sample before NMR data collection. The final concentration of RNA samples ranged between 0.5 and 1.0 mM.

#### Solid-phase synthesis

Unlabeled TAR and TAR^C24G^ were synthesized using a MerMade 12 Oligo Synthesizer (BioAutomation) using standard phosphoramidite chemistry and base and 2′-hydroxyl deprotection protocols as described previously [67]. Unlabeled phosphoramidites were purchased from ChemGenes. All RNA samples used 2′-TBDMS protected phosphoramidites with 1 μmol standard (1000 Å) synthesis columns. The 5′-DMT (4,4′-dimethoxytrityl) was removed for DMT-off deprotection and standard PAGE purification as previously described. Nucleobase protecting groups were removed and oligonucleotides were cleaved from the synthesis columns using 1 ml of 30% ammonium hydroxide and 30% methylamine (1:1) followed by incubation at room temperature for 2 h. Deprotected samples were then air-dried followed by addition of 100 μl DMSO and incubation at 65°C for 5 min to ensure the samples were fully dissolved. Then 125 μl of TEA-3HF was added and incubated at 65°C for 2.5 h. Finally, samples were precipitated overnight using 3 M sodium acetate and 100% ethanol, air-dried, then dissolved in water for gel purification, elution, ethanol washing, and buffer exchange as described for *in vitro* transcription earlier. The final concentration of unlabeled and site-labeled RNA NMR samples was ∼1.0 mM.

### NMR experiments

All NMR experiments were performed using the following Bruker spectrometers: an Avance III 600 MHz with a 5 mm triple-resonance cryoprobe, an Avance III 700 MHz with a triple-resonance HCN cryogenic probe, an Avance III 800 MHz with a triple-resonance TXO cryoprobe, an AVANCE-II 900 MHz with a 5 mm CPTCI H-C/N/D Z-GRD cryoprobe, and an AVANCE-NEO 900 MHz with a 5 mm CP2.1 TCI 900S6 H&F/C/N-D-05 Z XT cryoprobe. NMR data were analyzed using NMRPipe [68] and SPARKY [69]. All experiments were performed in NMR buffer with 15 mM sodium phosphate, 25 mM NaCl, 0.1 mM EDTA, and 10% D_2_O at 25°C, unless stated otherwise. All pH values refer to the bulk solution pH of the buffer, as measured with a glass electrode, which determines the overall proton activity of the solution.

#### Resonance assignments

NMR chemical shift assignments for TAR and TAR^ES2^ were obtained from prior studies [59, 60]. The resonance assignments for TAR^C24G^ (Supplementary Fig. S5A) were obtained from comparisons to the 2D HSQC spectra for TAR.

#### Off-resonance R_1ρ_ relaxation dispersion

Off-resonance ^13^C and ^15^N *R*_1ρ_ experiments were performed using a 1D scheme that uses selective Hartman–Hahn magnetization transfers as described previously [51]. Briefly, weakly matched ^1^H and ^13^C/^15^N RF fields were used to selectively transfer the magnetization from proton to carbon nucleus of interest. The longitudinal ^13^C/^15^N magnetization was allowed to relax for 5 ms to allow the substrates to equilibrate and then tilted along the effective field. A ^13^C spin-lock was applied for a maximal duration (≤120 ms) to obtain ∼70% decay in signal intensity at the end of the relaxation period. The signal intensity was recorded for five delays evenly spaced over the total relaxation period. Spin-lock powers, offsets, and delay times are summarized for each resonance in Supplementary Tables S5 and S6.

The *R*_1ρ_ measurements were performed on TAR at 25°C and pH = 5.4, 6.0, 6.4, 7.0, 7.4, and 8.4. For TAR^ES2^, the *R*_1ρ_ measurements were performed at 25°C and pH = 5.4, 6.0, and 6.4. In all cases, the NMR buffer consisted of 15 mM sodium phosphate, 25 mM NaCl, and 0.1 mM EDTA with 10% D_2_O.

#### Two-state analysis of *R*_1ρ_ data

The off-resonance *R*_1ρ_ data were analyzed as described previously [57]. Peak intensities were obtained using NMRPipe [68], and fitted to a mono-exponential decay as a function of the delay time to obtain the *R*_1ρ_ value for the various spin-lock power and offset combinations. The *R*_1ρ_ uncertainty was estimated using a Monte Carlo procedure as described previously [51]. *R*_1ρ_ values, measured as a function of various spin-lock power and offset combinations, were fit to a two-state exchange model using the Bloch–McConnell equations to extract exchange parameters of interest [70], including the population of the minor state (p_minor_), the exchange rate between the minor and dominant state (*k*_ex_ = *k*_forward_ + *k*_reverse_), the difference between the chemical shifts of the minor and dominant states (Δω = ω_minor_ - ω_dominant_), as well as the transverse (*R*_2_) and longitudinal (*R*_1_) relaxation rates. In all cases, the fit assumed that *R*_2,dominant _= *R*_2,minor _= *R*_2_ and *R*_1,dominant _= *R*_1,minor _= *R*_1_. The initial alignment of magnetization during the Bloch–McConnell simulations was determined based on the *k*_ex_/Δω ratio, as described previously [51]. The uncertainty in the exchange parameters was obtained using a Monte Carlo scheme as described previously [71].

Global fitting of the *R*_1ρ_ data across different nuclei was performed by sharing *k*_ex_ and *p*_ES2_. For TAR, the *R*_1ρ_ data for U38-N3, U23-C6, and A35-C8 were globally fitted at pH = 5.4–8.4. The fitted exchange parameters are summarized in Supplementary Table S1. For TAR^ES2^, the *R*_1ρ_ data for U38-N3 and U23-C6 were globally fitted at pH = 5.4–6.4. The fitted exchange parameters are summarized in Supplementary Table S2.

#### Constrained three-state global fit of *R*_1ρ_ data

The *R*_1ρ_ data measured for the TAR at pH = 5.4–7.4 were subject to a constrained three-state global fit assuming a linear topology (GS⇌GS^+^⇌ES2^+^). The pH-dependent populations were deduced based on the apparent p*K*_a_ ∼ 5.6 obtained from a global two-state fit of the *R*_1ρ_ data_,_ and the p*K*_a,ES2_ ∼ 4.0 obtained from pH-dependent CSP on the monomer [72]. The GS⇌GS^+^ exchange rate was fixed to *k*_ex,prot_ = *k*_prot_([H^+^] + 10^−p^*^K^*^a,GS^) in which *k*_prot_ was assumed to be ∼ 6 × 10^11^ M^−1^s^−1^ as observed for diffusion-limited protonation in prior studies [58, 73, 74]. The Δω values were allowed to vary with starting values (Supplementary Table S4) obtained as follows. Since U38-N3, U23-C6, and A35-C8 predominantly sense the conformational change between the GS and ES2, $\Delta {{\omega }_{GS\rightarrow GS + }} \approx 0\ ppm$ and $\Delta {{\omega }_{GS\rightarrow ES2 + }} = ( {\frac{1}{{1 - {{f}_{GS + /GS}}}}} )\Delta {{\omega }_{RD}} \approx \Delta {{\omega }_{RD}}$ as the fractional population ${{f}_{GS + /GS}} = \frac{{[ {G{{S}^ + }} ]}}{{[ {GS} ] + [ {G{{S}^ + }} ]}} \ll 1$. Here $\Delta {{\omega }_{RD}}$ represents the best-fit Δω obtained from a two-state fit of the *R*_1ρ_ profile. The *k*_ex,conf_, *R*_1,GS_ = *R*_1,GS+_ = *R*_1,ES2+_ and *R*_2,GS_ = *R*_2,GS+_ = *R*_2,ES2+_ were allowed to vary.

#### Explicit kinetic simulations

To capture the effect of ES1 on the observed pH-dependent kforward, we performed explicit kinetic simulations to model the three-state exchange (ES1^+^⇌GS⇌ES2^+^), as previously described [58]. An in-house Python script was used to stably solve the differential equations describing the time evolution of each individual species in the kinetic pathway. Input for the simulation included all microscopic rate constants for each step ($k_{on}^{ES1}[ {{{H}^ + }} ]$, $k_{off}^{ES1}$ for GS⇌ES1^+^ step and $k_{on}^{ES2}[ {{{H}^ + }} ]$, $k_{off}^{ES2}$ for GS⇌ES2^+^ step).The values for the microscopic rate constants were obtained from pH dependence of the *k*_forward_ and *k*_reverse_ (Fig. 7B). The microscopic rate constants were used to construct a rate matrix K that captures the three coupled kinetic differential equations:


\begin{eqnarray*}
&&\frac{d}{{dt}}\left[ {\begin{array}{@{}*{1}{c}@{}} {\left[ {ES{{1}^ + }} \right]\left( t \right)}\\ {\left[ {GS} \right]\left( t \right)}\\ {\left[ {ES{{2}^ + }} \right]\left( t \right)} \end{array}} \right] = K\left[ {\begin{array}{@{}*{1}{c}@{}} {\left[ {ES{{1}^ + }} \right]\left( t \right)}\\ {\left[ {GS} \right]\left( t \right)}\\ {\left[ {ES{{2}^ + }} \right]\left( t \right)} \end{array}} \right]\\&&\quad=\, \left[ {\begin{array}{@{}*{3}{c}@{}} { - k_{off}^{ES1}}&{k_{on}^{ES1}\left[ {{{H}^ + }} \right]}&0\\ {k_{off}^{ES1}}&{ - \left( {k_{on}^{ES1}\left[ {{{H}^ + }} \right] + k_{on}^{ES2}\left[ {{{H}^ + }} \right]} \right)}&{k_{off}^{ES2}}\\ 0&{k_{on}^{ES2}\left[ {{{H}^ + }} \right]}&{ - k_{off}^{ES2}} \end{array}} \right]\\&&\qquad\qquad\times\left[ {\begin{array}{@{}*{1}{c}@{}} {\left[ {ES{{1}^ + }} \right]\left( t \right)}\\ {\left[ {GS} \right]\left( t \right)}\\ {\left[ {ES{{2}^ + }} \right]\left( t \right)} \end{array}} \right].
\end{eqnarray*}

The matrix equation was solved by diagonalizing the rate matrix K as done in prior studies [75]. For the initial conditions, the population of the dominant species was set to 100%, while all other species initialized at 0%. To ensure the inferred kinetic rates were independent of initial conditions, multiple starting conditions were tested. The simulations were performed for varying time periods (0.01 ms to 0.1 ms) to ensure all species reached equilibrium. The time trace of ES2^+^ was fit to a standard mono-exponential decay curve to determine *p*_minor_ and *k*_ex_ of the overall proton-coupled conformational transition.

#### CEST analysis

^1^H CEST experiments were performed on Bruker AVANCE III 600 (TAR and TAR^C24G^ at pH 6.4) and 700 MHz spectrometers (TAR and TAR^C24G^ at pH 5.4 and 7.4). Data were collected at T = 25°C, unless stated otherwise, using a relaxation delay of 100 ms. The radiofrequency fields (RF, ω_1_2π^−1^) ranged between 10 and 1000 Hz, and the offsets (Ω2π^−1^) spanned ±6 ppm (Supplementary Table S7). The RF inhomogeneities were calibrated and accounted for during CEST fitting, as previously described [76]. The peak intensities at each RF power and offset were extracted using NMRPipe. The experimental uncertainty was obtained based on the standard deviation in peak intensities obtained from triplicate CEST experiments with zero relaxation delay for a given RF power. Exchange parameters for TAR and TAR^C24G^ (summarized in Supplementary Table S3) were determined by fitting the normalized intensities to a two-state Bloch–McConnell equation using an in-house Python script [76]. Errors in the exchange parameters correspond to the standard error (SEM), derived from the square root of the diagonal elements of the covariance matrix obtained from the fits. The ¹H CEST profiles were fitted both with and without exchange (*p*_ES_ = *k*_ex_ = Δω = 0) exchange. Model selection for fits with and without exchange was performed as previously described by computing Akaike (wAIC) and Bayesian information criterion (wBIC).

### Data analysis

The visualization of the TAR^ES2^ ensemble was carried out using PyMOL (https://pymol.org/). All bp geometries, hydrogen-bond, backbone, stacking, and sugar dihedral angles were calculated using X3DNA-DSSR [77]. Best-fit parameters for the thermodynamic models to pH-dependent ES2 population were obtained using non-linear least squares fitting using the “curve_fit” function in the SciPy Python package, starting from multiple initial conditions to ensure global convergence to the optimal solution. Note, throughout our thermodynamic and kinetic modeling, we refer to the p*K*_a_ of a specific conformational state (such as ES1 or ES2) as its “intrinsic” p*K*_a_, decoupled from any conformational changes, which incorporates the energetic contribution from additional hydrogen bonding upon protonation.

## Results

### Evidence TAR ES2 is protonated from pH-dependent *R*_1ρ_ measurements

To assess whether ES2 was protonated, we performed ^13^C and ^15^N *R*_1ρ_ relaxation dispersion (RD) experiments [78–80] on uniformly ^13^C/^15^N labeled TAR across a range of pH conditions. These experiments enable quantitative measurements of the kinetic rates governing the interconversion between the dominant GS and lowly populated ES, while also elucidating the chemical shifts of the minor ES, which serve as structural reporters. The experiments measure how the exchange contribution (*R*_ex_) to the transverse relaxation rate (*R*_2_) varies as a function of changing the spin lock power (ω_SL_) and frequency offset (ω_RF_) of a continuous radiofrequency field. The RD profiles are typically displayed by plotting the measured *R*_2 _+ *R*_ex_ as a function of ω_SL_ and ω_RF_. For detectable exchange between a GS and ES, a peak is observed centered at the difference between the chemical shifts of the major and minor states.

**Figure 2.**
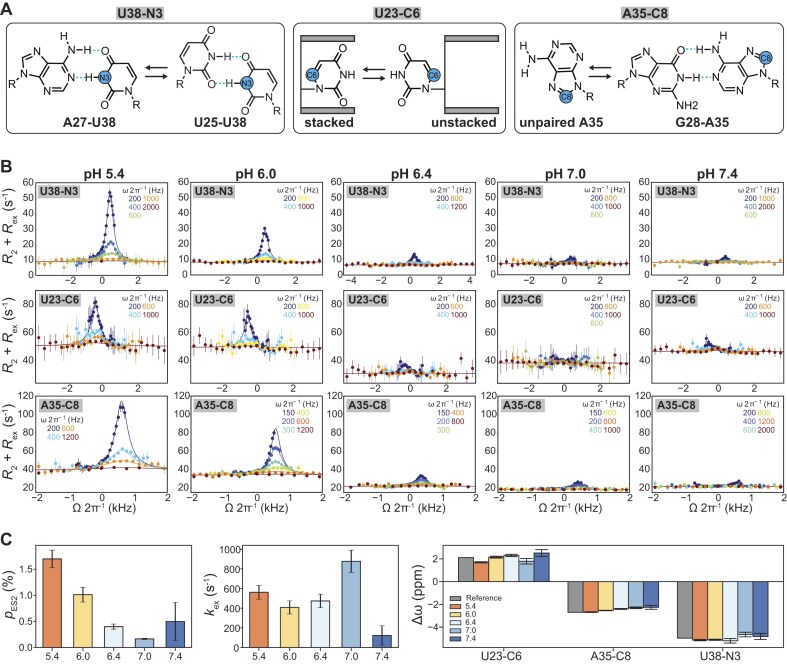
pH-dependent off-resonance *R*_1ρ_ measurements in TAR reveal ES2 is protonated. (**A**) Resonances in TAR used to probe the GS⇌ES2 exchange are highlighted in blue. (**B**) Off-resonance ^15^N and ^13^C *R*_1ρ_ relaxation dispersion profiles measured for U38-N3, U23-C6, and A35-C8 at pH = 5.4, 6.0, 6.4, 7.0, and 7.4. Spin-lock powers are color-coded. Solid lines represent the global two-state fits using the Bloch–McConnell equation (see the “Materials and methods” section). Error bars indicate ±1 standard deviation from Monte Carlo simulations (500 iterations per measurement). All NMR experiments were conducted in buffer containing 15 mM sodium phosphate, 25 mM NaCl, and 0.1 mM EDTA. Differences in the intrinsic *R*_2_ values for the ^13^C *R*_1ρ_ profiles across pH conditions are due to measurements being conducted at different magnetic fields ranging from 600 MHz to 900 MHz, as indicated in Supplementary Table S5. (**C**) The pH-dependent exchange parameters: population (*p*_ES2_), exchange rate (*k*_ex_ = *k*_forward_ + *k*_reverse_), and chemical shift difference (Δω = Δω_ES2_ − Δω_GS_) obtained from global fitting of the *R*_1ρ_ profiles at various pH. Δω_Reference_ (in gray) is the difference between the GS chemical shifts measured for TAR^ES2^ and TAR (Δω_Reference_ = ω_TARES2_ – ω_TAR_). The uncertainty in the fitted exchange parameters was estimated using a Monte Carlo approach (500 iterations) and is presented as mean ± 1 SD. The minor deviations observed for U23-C6 here and C24-C1′/C5 in Fig. 4 occur at large offset frequencies (Ω), where relaxation is dominated by *R*_1_ rather than *R*_2 _+ *R*_ex_, a common feature in such experiments [78].

By comparing RD profiles across pH, we assessed whether the GS⇌ES2 exchange is pH-dependent and thus coupled to protonation. We performed off-resonance *R*_1ρ_ experiments targeting the U38-N3, U23-C6, and A35-C8 nuclei, previously shown to be specifically sensitive to exchange involving ES2 and not ES1 (Fig. 2A). Briefly, residues U23 and U38 are >3 bp away from the TAR apical loop and hence do not sense the formation of ES1. In contrast, A35 in the apical undergoes changes in base pairing in both ES1 and ES2. However, prior RD measurements for A35-C8 suggest it only senses ES2, likely due to the higher *R*_ex_ contribution from ES2 compared to ES1, which has a much faster *k*_ex_ closer to the detection limit (∼30 000 s^−1^ for ES1 compared to ∼500 s^−1^ for ES2). Because these probes primarily sense conformational changes (Fig. 2A), not protonation itself, and do not distinguish between GS and ES1, they report on the apparent two-state equilibrium:

[GS + ES1 + ES1^+^] ⇌ [ES2 + ES2^+^].

Thus, what we refer to as the GS represents the combined contributions of GS + ES1 + ES1^+^ in fast exchange on the NMR chemical shift timescale. Conversely, ES2 represents the combined contributions of ES2 + ES2^+^ in fast exchange on the NMR chemical shift timescale.

Consistent with protonation-dependent exchange, all three probes exhibited a strong pH-dependent *R*_ex_ contribution, which increased with decreasing pH and became negligible at pH ≥ 7.4 (Fig. 2B and Supplementary Fig. S1). The absence of detectable exchange at higher pH suggests that the neutral ES2 species, if present, either exists at a population below the detection limit of RD or requires a protonated intermediate to produce exchange kinetics within the detectable timescale window. Note, the deviations in the observed kex at pH ≥ 7 likely reflect higher uncertainty in the best-fit exchange parameters due to significantly reduced *R*_ex_ contribution to the *R*_1ρ_ profiles compared to those at pH < 7.

The *R*_1ρ_ profiles for all three probes (U38-N3, U23-C6, and A35-C8) measured at pH = 5.4, 6.0, 6.4, 7.0, and 7.4 could be globally fit to a two-state (GS⇌ES2) exchange model indicating that they report on the same underlying exchange process. The global fit yielded the chemical shift differences between the ES2 and the GS (Δω = ω_ES2_ − ω_GS_), which were in excellent agreement across pH conditions, indicating that that same ES2 is detected over this pH range (Fig. 2C). The *R*_1ρ_ profiles provided no evidence for a third exchanging state.

As expected, if ES2 were protonated, the fitted ES2 population (*p*_ES2_) increased with lowering the pH by ∼10-fold from ∼0.17% at pH 7.0 to ∼1.70% at pH 5.4 (Fig. 2C), while the exchange rate (*k*_ex_ = *k*_forward_ + *k*_reverse_) decreased only ∼2-fold, from ∼877 s^−1^ to ∼562 s^−1^. At pH 7.4 and 8.4 *p*_ES2_ and *k*_ex_ could not be reliably determined due to a negligible *R*_ex_ contribution. Notably, since decreasing the pH should also favor GS = GS + ES1 + ES1^+^ by increasing the population of ES1^+^, the observed increase in the ES2 = ES2 + ES2^+^ population provides strong evidence that ES2 exists in a protonated form. Importantly, over the pH range 6.4–7.4, the increase in *p*_ES2_ was only two-fold, consistent with a depressed overall apparent p*K*_a_ and highlighting the importance of working under extreme acidic conditions to uncover this pH dependence.

### Evidence ES2 is protonated from pH-dependent *R*_1ρ_ measurements on TAR^ES2^

**Figure 3.**
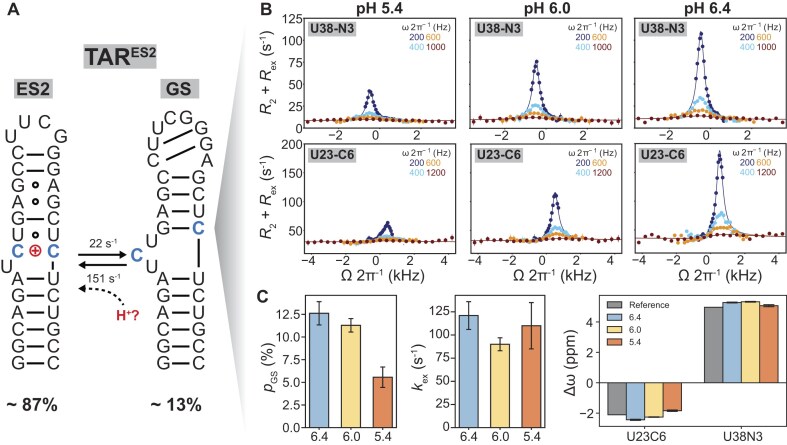
pH-dependent off-resonance *R*_1ρ_ measurements in TAR^ES2^ mutant support ES2 being protonated. (**A**) The TAR^ES2^ predominantly forms the ES2 conformation but also back-exchanges with a lowly populated and short-lived GS-like conformation at pH = 6.4 [10]. (**B**) Off-resonance ^15^N and ^13^C *R*_1ρ_ relaxation dispersion profiles measured for U38-N3 and U23-C6 at pH = 5.4, 6.0, and 6.4. Spin-lock powers are color-coded. Solid lines represent the global two-state fits using the Bloch–McConnell equation (see the “Materials and methods” section). Error bars indicate ±1 standard deviation from Monte Carlo simulations (500 iterations per measurement). All NMR experiments were conducted in buffer containing 15 mM sodium phosphate, 25 mM NaCl, and 0.1 mM EDTA. All experiments were conducted at an external field B_0_ = 900 MHz, as indicated in Supplementary Table S6. (**C**) The pH-dependent exchange parameters: population of the minor GS-like conformation (*p*_GS_), exchange rate (*k*_ex_ = *k*_1_ + *k*_-1_), and chemical shift difference (Δω = Δω_GS_ – Δω_ES2_) obtained from global fitting of the *R*_1ρ_ profiles at various pH. Δω_Reference_ (in gray) is the difference between the GS chemical shifts measured for TAR and TAR^ES2^ (Δω_Reference_ = ω_TAR_ – ω_TARES2_). The uncertainty in the fitted exchange parameters was estimated using a Monte Carlo approach (500 iterations) and is presented as mean ± 1 SD.

As an additional test of whether ES2 is protonated, we examined the pH dependence of ES2⇌GS exchange for a TAR mutant (TAR^ES2^)[10, 59], which preferentially stabilizes ES2 as the dominant state (Fig. 3A). TAR^ES2^ forms a structure closely mimicking that of ES2, as demonstrated by strong agreement between its chemical shifts and those of ES2 measured using NMR chemical exchange methods [10]. The TAR^ES2^ mutant inverts the conformational equilibrium between GS and ES2 observed in *wt* TAR, predominantly forming the ES2 conformation while undergoing back-exchange with a minor GS-like conformation with a population of ∼13% and a lifetime of ∼6 ms at pH = 6.4 as characterized by NMR relaxation dispersion measurements (Fig. 3A). Because TAR^ES2^ replaces the wild-type ES2 apical loop but preserves the G–A and C–C mismatches in the upper helix, which are the potential protonation sites (Figs 1A and 3A), we predicted that this back exchange process would exhibit an inverse dependence on pH relative to TAR: the now neutral minor state should increase in population with increasing pH and decrease with lowering pH.

Indeed, the U38-N3 and U23-C6 *R*_1ρ_ profiles measured for TAR^ES2^ exhibited a pH-dependence, which was inverted relative to TAR (Fig. 3B). The *R*_ex_ exchange contribution increased when increasing pH from 5.4 to 6.4, corresponding to a 2.3-fold increase in the population of the neutral GS-like conformation from 5.6 ± 1.1% to 12.6 ± 1.3% (Fig. 3B and C). Further increasing the pH to 7.4 resulted in substantial line broadening of resonances in the bulge and upper helix (Supplementary Fig. S2), most likely due to an increase in the exchange contribution. Because TAR^ES2^ retains the pH-dependent exchange despite replacing the wild-type ES2 apical loop, these results effectively rule out the apical loop residues as the primary site of protonation in ES2.

Taken together, the pH-dependent *R*_1ρ_ measurements on TAR as well as TAR^ES2^ provide strong evidence that the previously characterized ES2 is protonated at pH = 6.4 with the C24–C39 mismatch as the most likely protonation site.

### Evidence for a protonated C–C mismatch in ES2 from *R*_1ρ_ and NMR analysis of TAR^ES2^

**Figure 4.**
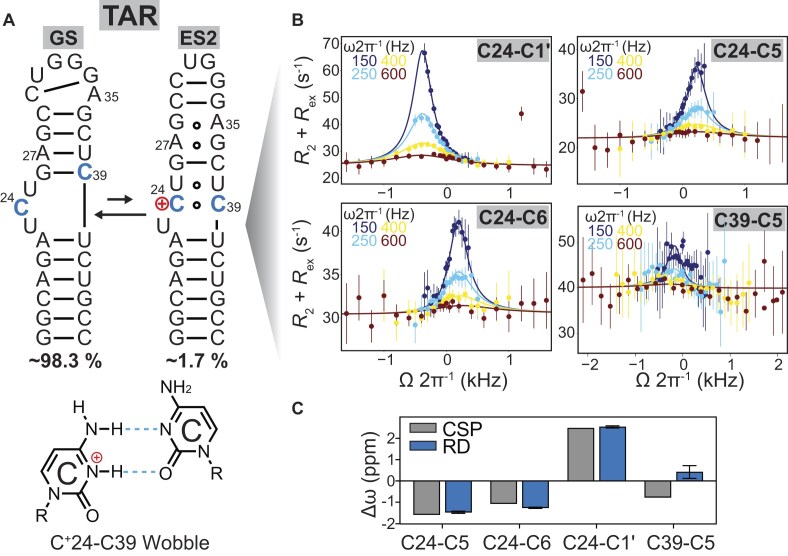
Evidence for a protonated C^+^24-C39 mismatch in ES2 from *R*_1ρ_ measurements on TAR. (**A**) Conformational exchange between the GS and ES2 in TAR at pH = 5.4. Also shown below is the chemical structure of the protonated C^+^24-C39 mismatch in the protonated ES2. (**B**) Off-resonance ^13^C *R*_1ρ_ relaxation dispersion profiles targeting the C24–C39 mismatch at pH = 5.4. Spin-lock powers are color-coded. Solid lines represent the global two-state fits using the Bloch–McConnell equation (see the “Materials and methods” section). Error bars indicate ±1 standard deviation from Monte Carlo simulations (500 iterations per measurement). All NMR experiments were conducted in buffer containing 15 mM sodium phosphate, 25 mM NaCl, and 0.1 mM EDTA at pH = 5.4. (**C**) The chemical shift difference (Δω = Δω_ES2_ – Δω_GS_) obtained from global fitting of the *R*_1ρ_ profiles at various pH. Δω_ref_ (in gray) is the difference between the GS chemical shifts measured for TAR^ES2^ and TAR (Δω = ω_TARES2_ – ω_TAR_). The uncertainty in the fitted exchange parameters was estimated using a Monte Carlo approach (500 iterations) and is presented as mean ± 1 SD.

In contrast to adenine, the commonly measured carbon and proton NMR chemical shifts on the cytosine nucleobase do not experience sizeable changes in chemical shifts that could be uniquely attributable to protonation of cytosine-N3. In particular, the cytosine-C5 carbon experiences a modest upfield shift of ∼1.0 ppm, while cytosine-C6 experiences a modest downfield shift of ∼1.6 ppm with cytosine-N3 protonation [53]. Nevertheless, to test the hypothesis that the C24–C39 mismatch is protonated in ES2, we performed additional *R*_1ρ_ experiments specifically targeting C24-C6, C5, as well as C39-C5, C1′ in TAR (Fig. 4A and B), which were not yet characterized by relaxation-dispersion measurements.

We worked under low pH = 5.4 to increase the ES2 population and enhance the reliability with which we can determine the Δω values for these resonances. We were unable to measure the *R*_1ρ_ profile for C39-C6 due to spectral overlap. The on-resonance *R*_1ρ_ profile measured for C39-C5 and C1′ exhibited a small to negligible exchange contribution, and HSQCs measured on TAR^ES2^ reveal minor chemical shift perturbations of ∼0.7 ppm for C39-C5 and ∼0 ppm for C39-C1′, implying that C39-N3 is not the primary protonation site (Supplementary Fig. S3A).

In contrast, the *R*_1ρ_ profiles for C24-C5 and C6 as well as C24-C1′ exhibited a strong *R*_ex_ contribution (Fig. 4B) and could be globally fitted to a two-state (GS⇌ES2) model, yielding *p*_ES2_ = 1.5 ± 0.1% and *k*_ex_ = 618 ± 58 s^−1^, in very good agreement with the values obtained independently using other probes (*p*_ES2_ = 1.7 ± 0.2% and *k*_ex_ = 562 ± 70 s^−1^). The global fit yielded Δω = 2.54 ± 0.05 ppm for C24-C1′, which was in excellent agreement with the Δω = 2.43 ± 0.06 ppm deduced at higher pH conditions and confirming that the *R*_1ρ_ profiles are detecting the same underlying ES2 across the different pH conditions. Notably, the Δω = −1.5 ppm for C24-C5 is consistent with protonation of this nucleobase when forming ES2 [53]. In contrast, the Δω = −1.2 ppm for C24-C6 likely reflects a combination of a downfield shift contribution due to protonation [53] and a larger upfield shift contribution due to the conformational transition from a bulge in the GS to the C^+^24-C39 mismatch in ES2, which is consistent with prior work [59, 81] showing that stacking of pyrimidine residues can result in larger chemical shift perturbations in C6 compared to C5. Furthermore, due to its unique structural context, a U25-U38 mismatch above and a 1 nt bulge below, which differs significantly from canonical Watson–Crick flanking bp, coupled with potential contributions from cation-pi interactions, could result in even larger stacking contributions to the observed C24-C6 Δω than those observed in prior studies [59, 81].

The excellent agreement between the Δω values for C24-C5, C6, and C1′ measured using *R*_1ρ_ and the values measured for the TAR^ES2^ mutant (Fig. 4C) support TAR^ES2^ as a conformational mimic of ES2, including the C24–C39 mismatch. Thus, we also re-examined NMR spectra of TAR^ES2^ at pH = 6.4 to obtain more direct evidence for the C24–C39 mismatch. Indeed, we observed a cross-peak in the 2D NOESY spectrum of TAR^ES2^, which could be assigned to C24⁺H3–U38H3 (Supplementary Fig. S3B). A similarly downfield-shifted C^+^H3 resonance was previously reported for the m¹G-C⁺ Hoogsteen bp [82], the C–G·C^+^ triplet [83], and in pH-dependent imino spectra of C–C mismatches in RNA duplexes [16]. In contrast, we did not observe any of the signatures expected for protonated G28-A35 or A27-G36 mismatches at pH = 6.4 (Supplementary Fig. S4A). Only when reducing the pH further to 5.4 did the TAR^ES2^ NMR spectra exhibit significant line broadening at A27-G36 and G28-A35, as well as the adjacent G26-C37 bp, most likely reflecting stabilization of the protonated G*_syn_*-A^+^ Hoogsteen conformation (Supplementary Fig. S4B), as expected given the lower p*K*_a_ ∼5.8–6.2 for the G–A mismatch [5, 23, 84, 85]. Taken together, these results suggest that at pH = 6.4, the C24–C39 mismatch is protonated, with C24 being the predominantly protonated cytosine.

### Engineering a TAR mutant with pH-independent ES2

**Figure 5.**
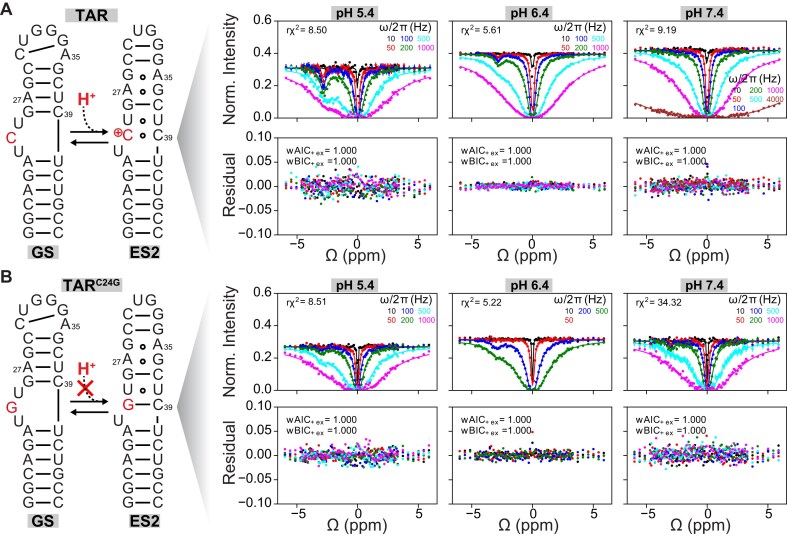
Engineered mutant decouples ES2 from protonation. (**A**) pH-dependent U38-H3 ¹H CEST profiles measured for GS⇌ES2 in TAR. (**B**) pH-dependent U38-H3 ¹H CEST profiles measured for the TAR^C24G^ mutant, which replaces the protonated C^+^24-C39 mismatch with a neutral G24-C30 Watson–Crick bp. The different RF powers applied are color-coded. All NMR experiments were conducted in buffer containing 15 mM sodium phosphate, 25 mM NaCl, and 0.1 mM EDTA. A two-state fit of the ¹H CEST profiles is shown as solid lines. Residual plots (measured normalized intensity – fit normalized intensity) are shown below each CEST profile. The corresponding reduced chi-square (rχ²), Akaike weight (wAIC), and Bayesian information criterion weight (wBIC) were used to evaluate the fit quality (see the “Materials and methods” section) and are shown within the CEST profiles. Error bars (often smaller than the data points) represent the standard deviation from triplicate CEST measurements of the peak intensity using zero relaxation delay collected at each RF field. Measurements at pH 6.4 for TAR and TAR^C24G^ were conducted on a 600 MHz spectrometer, whereas measurements at pH 5.4 and 7.4 were performed on a 700 MHz spectrometer, as indicated in Supplementary Table S7.

To further verify that C24–C39 is protonated in ES2, we designed a TAR mutant bearing the C24G mutation (TAR^C24G^), which replaces the proposed protonated C^+^24-C39 mismatch in ES2 (Fig. 5A) with a neutral G24-C39 Watson–Crick bp (Fig. 5B). Because this mutation minimally affects the stability of the GS, replacing the C24 bulge with G24, it was expected to retain the GS as the dominant conformation and to retain GS⇌ES2 exchange. If the C^+^24-C39 mismatch was indeed the site of protonation in ES2, we would expect that TAR^C24G^ should uncouple ES2 from protonation and undergo pH-independent GS⇌ES2 exchange (Fig. 5A and B). To test this prediction, we performed ^1^H CEST [76] experiments, which obviate the need for ^13^C/^15^N labeling. This experiment utilizes the guanine and uridine imino ^1^H resonances to measure conformational exchange. Like the *R*_1ρ_ experiment, the ^1^H CEST profiles can be fit to a two-state GS⇌ES2 exchange model to obtain the *p_ES_* and *k*_ex_, along with Δω for each targeted imino proton.

As a positive control, we measured ^1^H CEST profiles for TAR at pH 5.4, 6.4, and 7.4, targeting the U38-H3 imino proton, which strongly senses the GS⇌ES2 exchange due to the formation of the A27-U38 Watson–Crick bp in the GS but a U25-U38 mismatch in ES2. At pH = 6.4, we observed the characteristic dip in the U38-H3 CEST profile at Δω ≈ –2.8 ppm, corresponding to the chemical shift of the U25-U38 mismatch in the minor ES2 state (Fig. 5A). Fitting the U38-H3 CEST profile to a two-state GS⇌ES2 exchange model yielded an ES2 population of ∼0.4% and *k*_ex_ ∼ 470 s^−1^, in excellent agreement with the values obtained using *R*_1ρ_ (population 0.4% and *k*_ex _= 474 s^−1^). As expected, reducing the pH from 6.4 to 5.4 increased the intensity of the dip, corresponding to a four-fold increase in the ES2 population from ∼0.4% at pH = 6.4 to ∼1.6% at pH = 5.4, in excellent agreement with the values obtained using *R*_1ρ_ (Fig. 5A). In contrast, increasing the pH from 6.4 to 7.4 resulted in a decrease in the intensity of the dip, with a fitted population of <0.1%, representing a >4-fold decrease in population relative to pH 6.4 (Fig. 5A).

NMR spectra of TAR^C24G^ confirmed that it adopts the GS as the dominant conformation (Supplementary Fig. S5A). Like for TAR, the U38-H3 CEST profile measured for TAR^C24G^ at pH = 6.4 also exhibited the characteristic dip at Δω ≈ –3 ppm, demonstrating that TAR^C24G^ also undergoes exchange with ES2 (Fig. 5B) and that the C24G mutation does not perturb the other mismatches in ES2. A two-state fit of the ^1^H CEST profile yielded a population of 0.3% and *k*_ex _= 864 s^−1^, very similar to that measured for TAR. These results suggest that the C24G substitution similarly modulates the energetics of the GS and ES2, thereby maintaining a comparable *p*_ES2_ value. While the G-C Watson–Crick bp is not isosteric with a C–C mismatch, for 60% of the conformations in the FARFAR-NMR-MD ensemble, the C–C mismatch adopted an unpaired Watson–Crick-like geometry, which could be mimicked by the G-C bp, thereby leading to similar stabilities.

In sharp contrast to TAR, the U38-H3 CEST profile measured for the TAR^C24G^ showed minimal changes when either lowering the pH to 5.4 or increasing it to 7.4 (Fig. 5B and Supplementary Fig. S5B). A two-state fit of the ^1^H CEST profile yielded an ES2 population of ∼0.3% at both pH 5.4 and ∼0.5% at pH 7.4, which was within error of the population measured at pH 6.4 (∼0.3%) and in excellent agreement with our predictions (Fig. 5B and Supplementary Fig. S5C).

Taken together, these results provide concrete evidence that the formation of the protonated C24–C39 drives the pH-dependent stabilization of the TAR ES2 conformation.

### Resolving intrinsic p*K*_a_ and conformational equilibria in a multi-state protonated ensemble

Elucidating the degree to which ES2 is energetically coupled to protonation requires determining the conformational equilibrium, ${\boldsymbol{K}}_{{\boldsymbol{conf}}}^{{\boldsymbol{ES}}2}$, describing the propensity of the GS to form the neutral ES2, as well as the intrinsic p*K*_a_^ES2^ describing the proton affinity of ES2:

#### GS ⇌ ES2 ⇌ ES2^+^


\begin{eqnarray*}
\frac{{{{{\left[ {{\boldsymbol{ES}}2} \right]}}_{{\boldsymbol{eq}}}}}}{{{{{\left[ {{\boldsymbol{GS}}} \right]}}_{{\boldsymbol{eq}}}}}} = {\boldsymbol{K}}_{{\boldsymbol{conf}}}^{{\boldsymbol{ES}}2},
\end{eqnarray*}

\begin{eqnarray*}
\frac{{{{{\left[ {{\boldsymbol{ES}}{{2}^ + }} \right]}}_{{\boldsymbol{eq}}}}}}{{{{{\left[ {{\boldsymbol{ES}}2} \right]}}_{{\boldsymbol{eq}}}}}} = {{10}^{{\boldsymbol{pK}}_{\boldsymbol{a}}^{{\boldsymbol{ES}}2} - {\boldsymbol{pH}}}},
\end{eqnarray*}

(1) \begin{eqnarray*}
\frac{{{{{\left[ {{\boldsymbol{ES}}{{2}^ + }} \right]}}_{{\boldsymbol{eq}}}}}}{{{{{\left[ {{\boldsymbol{GS}}} \right]}}_{{\boldsymbol{eq}}}}}} = {\boldsymbol{K}}_{{\boldsymbol{conf}}}^{{\boldsymbol{ES}}2} \times {{10}^{{\boldsymbol{pK}}_{\boldsymbol{a}}^{{\boldsymbol{ES}}2} - {\boldsymbol{pH}}}}.
\end{eqnarray*}

In which ${{[ {{{\bf GS}}} ]}_{{{\bf eq}}}}$, ${{[ {{{\bf ES}}2} ]}_{{{\bf eq}}}}$, and ${{[ {{{\bf ES}}{{2}^ + }} ]}_{{{\bf eq}}}}{\mathrm{\ }}$are the equilibrium populations of the neutral GS and the neutral and protonated ES2 species, and in which we assume a Hill coefficient *n *= 1. For a proton-coupled conformational transition, the measured apparent p*K*_a_ (${\boldsymbol{pK}}_{\boldsymbol{a}}^{{\boldsymbol{apparent}}}$) will be the intrinsic ${\boldsymbol{pK}}_{\boldsymbol{a}}^{{\boldsymbol{ES}}2}\ $modified by a contribution representing the energetic penalty to form the neutral ES2 starting from the neutral GS.


(2) \begin{eqnarray*}
{\boldsymbol{pK}}_{\boldsymbol{a}}^{{\boldsymbol{apparent}}} = {\boldsymbol{pK}}_{\boldsymbol{a}}^{{\boldsymbol{ES}}2} + {{\log }_{10}}{\boldsymbol{K}}_{{\boldsymbol{conf}}}^{{\boldsymbol{ES}}2}.\
\end{eqnarray*}

Without independent knowledge of ${\boldsymbol{K}}_{{\boldsymbol{conf}}}^{{\boldsymbol{ES}}2}$ it is not feasible to deduce the intrinsic p*K*_a_^ES2^ of ES2. A higher p*K*_a_ could be compensated for by a lower *K*_conf_ leading to the same apparent p*K*_a_ as clearly observable in equation (2).

**Figure 6.**
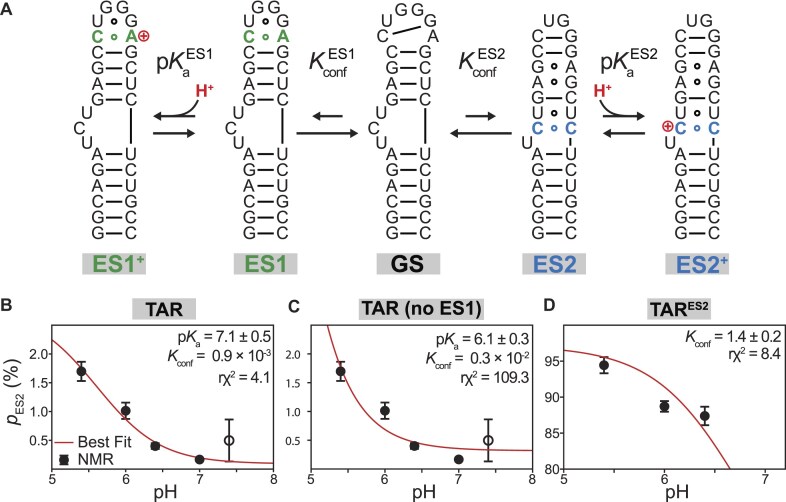
Multi-state protonated ensemble analysis of pH-dependent apparent TAR ES2 population. (**A**) Five-state thermodynamic model with neutral GS, ES1, and ES2 along with the protonated states ES1^+^ and ES2^+^. The neutral ES1 and ES2 are related to the neutral GS by their corresponding conformational equilibria ($K_{\textit{conf}}^{ES1}$, $K_{\textit{conf}}^{ES2}$) while the protonated ES1^+^ and ES2^+^ are related to their neutral species ES1 and ES2, respectively, by their intrinsic p*K*_a_  $pK_a^{ES1}$ and $pK_a^{ES2}$, respectively. (**B**–**D**) Best-fit five-state thermodynamic model of the pH-dependent ES2 population (*p*_ES2_) for (B) TAR and (C) TAR^ES2^ obtained using least-squares curve fitting (see the “Materials and methods” section). Also shown in panel (D) is the best-fit model for TAR without incorporating the contribution from ES1 in the overall pH-dependent apparent ES2 population, showing worse fit compared to that including the ES1 (Fig. 6B). Shown in all three plots are the pH-dependent apparent *p*_ES2_ comprising both the neutral (ES2) and protonated (ES2^+^) species as obtained from *R*_1ρ_ measurements (black circle) along with the predictions (red line) from the best-fit thermodynamic models. The fit values of the thermodynamic parameters (intrinsic $pK_a^{ES2}$, $K_{\textit{conf}}^{ES2}$) as well as the reduced χ^2^ quantifying the quality of the fits are shown on the upper right corner. For TAR^ES2^, the intrinsic $pK_a^{ES2}$ was set to the value obtained for TAR of ∼7.1. For TAR, the *p*_ES2_ determined from pH = 7.4 (hollow symbol) was not included for fitting, owing to high errors in the estimated *p*_ES2_ and *k*_ex_ due to the relatively minor *R*_ex_ contribution in the *R*_1ρ_ profile.

The situation is more complicated in the case of TAR because there are two distinct protonated species, which sets up a multi-state system with coupled thermodynamic equilibria (Fig. 6A):

#### ES1^+^ ⇌ ES ⇌ GS ⇌ ES2 ⇌ ES2^+^

As noted previously, our NMR probes reporting on the apparent two-state exchange process:

[GS + ES1 + ES1^+^] ⇌ [ES2 + ES2^+^]

Similar to ES2, the conformational equilibrium, ${\boldsymbol{K}}_{{\boldsymbol{conf}}}^{{\boldsymbol{ES}}1}$, describing the propensity of the GS to form the neutral ES1, as well as the intrinsic p*K*_a_^ES1^ describing the proton affinity of ES1, are defined as:


\begin{eqnarray*}
\frac{{{{{\left[ {{\boldsymbol{ES}}1} \right]}}_{{\boldsymbol{eq}}}}}}{{{{{\left[ {{\boldsymbol{GS}}} \right]}}_{{\boldsymbol{eq}}}}}} = {\boldsymbol{K}}_{{\boldsymbol{conf}}}^{{\boldsymbol{ES}}1},
\end{eqnarray*}

\begin{eqnarray*}
\frac{{{{{\left[ {{\boldsymbol{ES}}{{1}^ + }} \right]}}_{{\boldsymbol{eq}}}}}}{{{{{\left[ {{\boldsymbol{ES}}1} \right]}}_{{\boldsymbol{eq}}}}}} = {{10}^{{\boldsymbol{pK}}_{\boldsymbol{a}}^{{\boldsymbol{ES}}1} - {\boldsymbol{pH}}}},
\end{eqnarray*}

(3) \begin{eqnarray*}
\frac{{{{{\left[ {{\boldsymbol{ES}}{{1}^ + }} \right]}}_{{\boldsymbol{eq}}}}}}{{{{{\left[ {{\boldsymbol{GS}}} \right]}}_{{\boldsymbol{eq}}}}}} = {\boldsymbol{K}}_{{\boldsymbol{conf}}}^{{\boldsymbol{ES}}1} \times {{10}^{{\boldsymbol{p}}K_{\boldsymbol{a}}^{{\boldsymbol{ES}}1} - {\boldsymbol{pH}}}}.
\end{eqnarray*}

The intrinsic p*K*_a_ of the C–A^+^ mismatch in ES1 (${\boldsymbol{pK}}_{\boldsymbol{a}}^{{\boldsymbol{ES}}1}$) is ∼7.5 from prior pH-dependent NMR kinetic measurements [58], and that of the C–C mismatch in ES2 (${\boldsymbol{pK}}_{\boldsymbol{a}}^{{\boldsymbol{ES}}2}$) is expected to fall in the range of ∼6.7–7.0 based on prior NMR pH-titrations of C–C mismatches [55, 65]. As a result, the populations of both ES1^+^ and ES2^+^ are predicted to be significant and to vary considerably over the pH range used for the NMR measurements, as highlighted in equations (1) and (3). Deducing the desired ${\boldsymbol{K}}_{{\boldsymbol{conf}}}^{{\boldsymbol{ES}}2}$ and p*K*_a_^ES2^ from these pH-dependent *p*_ES2_ measurements requires a thermodynamic model that accounts for the pH-dependent changes in the equilibrium populations of all five states: GS, ES1, ES1^+^, ES2, and ES2^+^. Note that equations (1)–(3) correspond to a purely thermodynamic decomposition of the proton-coupled conformational equilibrium in TAR, independent of the dominant kinetic pathway for this transition, which holds true even if the dominant kinetic pathway does not proceed via the pathway involving conformational change prior to protonation.

The apparent *p*_ES2_, comprising both the protonated and neutral ES2 conformations, as measured by NMR, is given by:


\begin{eqnarray*}
{{{\boldsymbol{p}}}_{{\boldsymbol{ES}}2}} = \frac{{{{{\left[ {{\boldsymbol{ES}}2} \right]}}_{{\boldsymbol{eq}}}} + {{{\left[ {{\boldsymbol{ES}}{{2}^ + }} \right]}}_{{\boldsymbol{eq}}}}}}{{{{{\left[ {{\boldsymbol{GS}}} \right]}}_{{\boldsymbol{eq}}}} + {{{\left[ {{\boldsymbol{ES}}1} \right]}}_{{\boldsymbol{eq}}}} + {{{\left[ {{\boldsymbol{ES}}{{1}^ + }} \right]}}_{{\boldsymbol{eq}}}} + {{{\left[ {{\boldsymbol{ES}}2} \right]}}_{{\boldsymbol{eq}}}} + {{{\left[ {{\boldsymbol{ES}}{{2}^ + }} \right]}}_{{\boldsymbol{eq}}}}}},
\end{eqnarray*}

(4) \begin{eqnarray*}
{{{\boldsymbol{p}}}_{{\boldsymbol{ES}}2}} = \frac{{{\boldsymbol{K}}_{{\boldsymbol{conf}}}^{{\boldsymbol{ES}}2}\left( {1 + {{{10}}^{{\boldsymbol{pK}}_{\boldsymbol{a}}^{{\boldsymbol{ES}}2} - {\boldsymbol{pH}}}}} \right)}}{{1 + {\boldsymbol{K}}_{{\boldsymbol{conf}}}^{{\boldsymbol{ES}}1}\left( {1 + {{{10}}^{{\boldsymbol{pK}}_{\boldsymbol{a}}^{{\boldsymbol{ES}}1} - {\boldsymbol{pH}}}}} \right) + {\boldsymbol{K}}_{{\boldsymbol{conf}}}^{{\boldsymbol{ES}}2}\left( {1 + {{{10}}^{p{\boldsymbol{K}}_{\boldsymbol{a}}^{{\boldsymbol{ES}}2} - {\boldsymbol{pH}}}}} \right)}}.\\
\end{eqnarray*}

Note that while we focus on the protonation of residues A35 and C24/C39 in our thermodynamic analysis, in principle, other residues in TAR that either form Watson–Crick bps or are unpaired can also undergo protonation. The p*K*_a_ of such Watson–Crick bp or unpaired residues is typically low in the range of ∼3.5–4.0. The low p*K*_a_, combined with the fact that their protonation does not lead to formation of stable protonated mismatches (such as C^+^–C or A^+^-C with elevated p*K*_a_ ∼6–7) in the TAR ensemble (GS, ES1, or ES2), implies that their contribution to the observed pH dependence of the GS, ES1, and ES2 populations is negligible compared to the contributions from the protonation of A35 and C24/C39 residues, which form the C30-A^+^35 and C^+^24-C39/C24-C^+^39 mismatches in ES1 and ES2, respectively. In addition, we recently dissected the kinetic mechanism of the proton-coupled GS⇌ES1^+^ conformational transition, from which we deduced ${\boldsymbol{pK}}_{\boldsymbol{a}}^{{\boldsymbol{ES}}1}$ ∼7.5 and ${\boldsymbol{K}}_{{\boldsymbol{conf}}}^{{\boldsymbol{ES}}1}$ ∼0.01. Here it is important to note that the exceptionally small population of ES2 (∼0.4%) makes it sensitive to even the presence of other protonated conformational states such as ES1 in the ensemble (as demonstrated in Fig. 6C), which forms with a much higher population of ∼15%. In contrast, the pH dependence of ES2 has minimal impact (below the detection limits of our NMR measurements) on the populations of ES1 inferred in prior studies due to the significantly smaller overall population of ES2 compared to that of ES1.

Fixing ${\boldsymbol{pK}}_{\boldsymbol{a}}^{{\boldsymbol{ES}}1}$ ∼7.5 and ${\boldsymbol{K}}_{{\boldsymbol{conf}}}^{{\boldsymbol{ES}}1}$ ∼0.01, we fit the pH-dependent *p*_ES2_ to Equation (4) using non-linear least-squares optimization over the pH range of 5.4 to 7.0. During the fit, the intrinsic $pK_a^{ES2}$ and $K_{\textit{conf}}^{ES2}$ were treated as free parameters.

The pH-dependent *p*_ES2_ could be satisfactorily fit using our thermodynamic model (Fig. 6B). In contrast, the agreement deteriorated when using a model that ignores the ES1^+^ contribution (Fig. 6C). The best-fit intrinsic $pK_a^{ES2}$ ∼7.1 ± 0.5 and $pK_a^{ES2}$ ≥ 6.4 deduced from a degeneracy analysis (Supplementary Fig. S6A and B) was consistent with values (p*K*_a_ = 6.7–7.0) previously reported for C–C mismatches in RNA and DNA duplexes [55, 65]. Note, the p*K*_a_ of C24-N3 in ES2 is expected to be significantly elevated compared to that of an unpaired cytosine (p*K*_a_ ∼4), reflecting the additional energetic stability due to formation of an extra hydrogen bond in the C^+^24-C39 mismatch. Based on $pK_a^{ES2}$ ≥ 6.4, ∼50%–80% of ES2 is protonated at pH = 6.4, which is in reasonable agreement with the ∼20% protonation-competent C24-C39 wobble conformations observed independently in the TAR^ES2^ FARFAR-MD-NMR ensemble [10]. We also verified that the alternative neutral conformations of the C–C mismatch were indeed needed to satisfy the RDCs measured in TAR^ES2^ at pH 6.4. Restricting the ensemble to only the protonation-competent wobble conformations significantly worsened the RDC agreement, increasing the RMSD from 2.7 Hz (original library) to 4.7 Hz (Supplementary Fig. S6C). Taken together, these results indicate that at pH 6.4, the C–C mismatch in ES2 exists in a near 50:50 dynamic equilibrium between protonated wobble and other neutral conformational states.

The best-fit $K_{\textit{conf}}^{ES2}$ ∼0.9 × 10^−3^ implies a high conformational penalty $\Delta G_{\textit{penalty}}^{ES2}\ \sim $4.1 ± 0.6 kcal/mol to form ES2, which is to be expected given replacement of several canonical Watson–Crick bps with non-canonical mismatches. Accordingly, our thermodynamic model predicts that at high pH (≥7.4), the neutral ES2 population is only ∼0.2%. Since the 0.4% ES2 population at pH = 6.4 was already near the edge of detection, this lower population, combined with a reduced exchange rate with increasing pH, likely accounts for the absence of detectable ES2 exchange in our *R*_1ρ_ experiments under these basic conditions (Supplementary Fig. S6D).

The high conformational penalty associated with the exceptionally lowly populated ES2 gives rise to a low apparent p*K*_a_ for the overall GS⇌ES2^+^ transition, $pK_a^{\textit{apparent}}$∼4.1 ± 0.7, significantly reduced from the conformation-specific p*K*_a_ (ES2⇌ES2^+^) of ES2 ∼6.4 observed in our thermodynamic model. This low $pK_a^{\textit{apparent}}$ partly explains why a significant reduction in pH was needed to observe a significant change in the *p*_ES2_ population. These findings underscore the challenges in probing protonation events in sparsely populated states and the added complexity that arises when multiple protonated species are present.

We also applied our thermodynamic model to analyze the pH-dependent *p*_ES2_ measured for the TAR^ES2^ mutant. If TAR^ES2^ is indeed an energetic mimic of ES2, we would expect it to recapitulate $pK_a^{ES2}$ ∼7.1 ± 0.5. TAR^ES2^ also eliminates ES1, simplifying analysis of the pH-dependence of *p*_ES2_:


(5) \begin{eqnarray*}
{{p}_{ES2}} = \frac{{{{{\left[ {ES2} \right]}}_{eq}} + {{{\left[ {ES{{2}^ + }} \right]}}_{eq}}}}{{{{{\left[ {GS} \right]}}_{eq}} + {{{\left[ {ES2} \right]}}_{eq}} + {{{\left[ {ES{{2}^ + }} \right]}}_{eq}}}}.
\end{eqnarray*}

Indeed, we were able to satisfactorily fit the pH-dependent *p*_ES2_ measured for TAR^ES2^ when fixing $pK_a^{ES2}$ ∼7.1 ± 0.5 to the value deduced for TAR (Fig. 6D). Again, a degeneracy analysis reveals $pK_a^{ES2}$ ≥ 6.4 (Supplementary Fig. S6B). Furthermore, the best-fit $K_{\textit{conf}}^{ES2}$ ∼1.4 ± 0.2 for TAR^ES2^ was ∼2300-fold higher than the corresponding value deduced for TAR, implying that the stable UUCG tetraloop preferentially stabilizes ES2 relative to the GS by ∼4.6 ± 0.4 kcal/mol, independent of pH.

Strikingly, $K_{\textit{conf}}^{ES2}$ ∼1.4 predicts that for TAR^ES2^, ${{[ {GS} ]}_{eq}}\sim {{[ {ES2} ]}_{eq}}$ independent of pH. This explained the independent observation of substantial broadening of resonance in TAR^ES2^ at elevated pH = 7.4, which can be attributed to significant line-broadening due to exchange between the two highly populated neutral species GS⇌ES2 (Supplementary Fig. S2). Furthermore, the high $pK_a^{ES2}$ ∼7.1 ± 0.5 also explains why no significant chemical shift perturbations were observed in TAR^ES2^ when lowering the pH from 6.4 to 5.4: under both pH conditions, the protonated ES2^+^ dominates while the population of neutral ES2 remains below 20% (Supplementary Fig. S4).

### An induced-fit kinetic mechanism with rate-limiting conformational change

**Figure 7.**
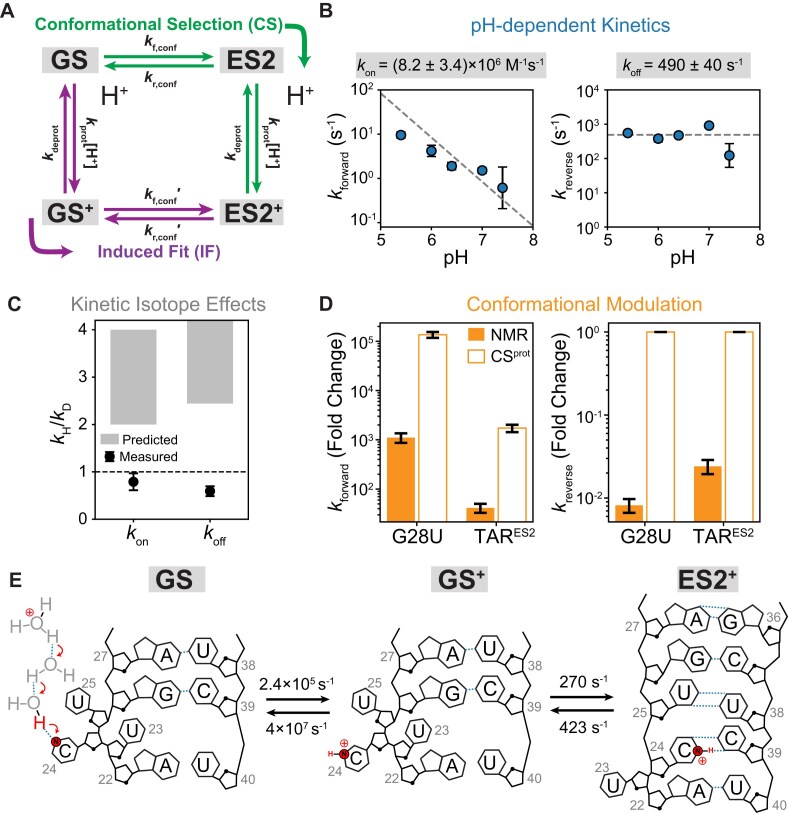
ES2 protonation occurs via an induced-fit pathway in which conformational change is rate limiting. (**A**) Four-state reaction mechanism underlying the proton-coupled conformational transition to form ES2 in TAR. *k*_f,conf_ and *k*_r,conf_ are the forward and reverse rate constants for the conformational exchange between neutral species, respectively; *k*_f,conf_′ and *k*_r,conf_′ are the forward and reverse rate constants for the conformational exchange between protonated species, respectively; *k*_prot_ is the second-order rate constant for protonation for both GS and ES2 conformations; and *k*_deprot,GS_ and *k*_deprot,ES2_ are the first-order rate constants for deprotonation for GS^+^ and ES2^+^, respectively. (**B**) Forward (*k*_forward_) and reverse (*k*_reverse_) rates obtained from a global two-state fit of the off-resonance *R*_1ρ_ profiles measured for U38-N3, U23-C6, and A35-C8 at varying pH. Error bars are presented as values ±1 SD from Monte Carlo simulations (number of iterations = 500) for one *R*_1ρ_ measurement as previously described. [51] Fitting *k*_forward_ and *k*_reverse_ using *k*_forward_ = *k*_on_[H^+^] and *k*_reverse_ = *k*_off_ yields *k*_on_ ∼ (8.2 ± 3.4) × 10^6^ M^−1^s^−1^ and *k*_off_ ∼ 490 ± 40 s^−1^. Note the deviations from linearity of the *k*_forward_ at lower pH could arise from additional contributions to pH-dependent kinetics from G–A mismatches or from potential contributions from the increasing ES1^+^, which could have slightly different ES2 exchange kinetics relative to GS. (**C**) Solvent KIEs for the proton-coupled ES2 conformational transition in TAR, in which H_2_O was replaced with 100% D_2_O at pH/pD = 6.4(60). The gray boxes denote the predicted range of *k*_H_/*k*_D_ values based on ∼2-to-4-fold increase in *k*_prot_ [99] and the observed ∼0.1 unit increase in intrinsic p*K*_a_ in deuterium [88], based on a kinetic mechanism where protonation is rate-limiting (either CS^prot^ or IF^prot^). Measured values of *k*_H_/*k*_D_ are shown as solid black circles. (**D**) Comparison of measured versus predicted fold changes in *k*_forward_ and *k*_reverse_ for the GS⇌ES2 exchange in TAR mutants: TAR^ES2^ and G28U. Both TAR^ES2^ and G28U introduce mutations that stabilize the lowly populated ES2 in TAR, rendering it the dominant state and significantly perturbing the conformational equilibrium by ∼10^3^-fold and ∼10^5^-fold, respectively. Predicted fold changes (open bars) were obtained assuming the CS^prot^ mechanism, where ${{k}_{\textit{forward}}} = \frac{{K_{\textit{conf}}^{ES2}}}{{K_{\textit{conf}}^{ES2} + 1}}{{k}_{\textit{prot}}}[ {{{H}^ + }} ]$ and ${{k}_{\textit{reverse}}} = {{k}_{\textit{deprot}}}$. (**E**) Proposed induced-fit mechanism for the proton-coupled conformational transition from GS to ES2. The populations of the neutral GS (∼99%), protonated GS^+^ (∼0.6%), and protonated ES2^+^ (∼0.4%) states, as well as the protonation rate ∼2.4 × 10^5^ s^−1^ at pH 6.4, were estimated using the apparent p*K*_a_ ∼ 4.1 and *k*_forward_ = *k*_prot_[H^+^] = 7 × 10^11^ M^−1^s^−1^ × (10^−pH^ M). The forward and reverse rate constants of conformational change correspond to the global minimum obtained from a three-state fit of the *R*_1ρ_ data (see the main text and “Materials and methods” section).

The protonated ES2^+^ could form via an induced-fit (IF) mechanism in which the exposed C24 bulge residue in the GS is initially protonated to form a GS^+^ intermediate, followed by a conformational change to form the protonated C^+^–C mismatch in ES2 (Fig. 7A). In our kinetic models, we refer to the intermediate species with a protonated C24 residue while maintaining the GS secondary structure as GS^+^. Alternatively, the conformational change could occur first to form a neutral ES2 intermediate, followed by protonation of the neutral C–C mismatch through a conformational selection (CS) mechanism (Fig. 7A). Furthermore, in each of these two pathways, either the conformational change (IF^conf^ and CS^conf^) or the protonation step (IF^prot^ and CS^prot^) could be rate-limiting (Fig. 7A). Despite their exquisite sensitivity to sparsely populated and short-lived species, these fleeting intermediates are not directly resolvable in the *R*_1ρ_ experiments, and all off-resonance *R*_1ρ_ profiles did not detect a third state. Alternative approaches are thus needed to dissect these reaction mechanisms.

Recently, we introduced an approach [58] to dissect the kinetic mechanisms of the proton-coupled conformational transitions. The approach uses changes in pH, chemical modifications, and solvent KIEs to perturb the conformational change and protonation steps independently, and NMR chemical exchange-based methods to measure the consequences of these perturbations on *k*_forward_ and *k*_reverse_ obtained from two-state fits of the data. When applied to TAR ES1, the approach revealed a reaction that follows an induced-fit mechanism, whereby the unpaired A35 in the GS is initially protonated, followed by rate-limiting intra-helical flipping to form the protonated C30-A^+^35 mismatch. In general, the kinetics of proton-coupled conformational transitions involving multiple protonated states cannot be predicted by such a simplified three-state approach, instead requiring numerical simulations to incorporate the contributions from each protonated state. However, the GS⇌ES1^+^ exchange (*k*_ex_ ∼25 000 s^−1^) occurs ∼2 orders of magnitude faster than the GS⇌ES2^+^ exchange (*k*_ex_ ∼500 s^−1^), and the probes used to detect ES2^+^ do not distinguish between the GS and ES1^+^. This further simplifies the analysis: because the GS⇌ES2^+^ exchange occurs on the much slower millisecond timescale, ES1^+^ rapidly achieves its equilibrium population, only contributing to the apparent GS population, without significantly impacting the exchange kinetics.

To dissect the kinetic mechanism of ES2 protonation, we first examined the pH dependence of *k*_forward_ and *k*_reverse_ for ES2 exchange measured for TAR using NMR *R*_1ρ_ at five different pHs ranging between 5.4 and 7.4. Consistent with *k*_forward_ = *k*_on_ [H^+^], the *k*_forward_ increased linearly with increasing [H^+^] (Fig. 7B). This result immediately ruled out conformational selection as the dominant pathway in which conformational change is the rate-limiting step (CS^conf^), since this mechanism predicts that *k*_forward_ remains unchanged with pH [58]. A linear fit of *k*_forward_ versus [H^+^] yielded a slope corresponding to a very slow *k*_on_ = (8.2 ± 3.4) $ \times $ 10^6^ M^−1^s^−1^, four to five orders of magnitude slower than diffusion-limited proton transfer *k*_prot_ ∼10^10^ - 10^11^ M^−1^s^−1^ deduced for the proton shuttling along hydrogen-bonded water molecules [73]. The slow *k*_on_ rules out an induced-fit mechanism in which protonation is the rate-limiting step (IF^prot^), since such a mechanism predicts a diffusion-limited *k*_on_ = *k*_prot_ ∼10^10^ - 10^11^ M^−1^s^−1^. Note, the small deviation from linearity for *k*_forward_ observed at lower pH ∼5.4–6.4 could be explained by the effect of GS⇌ES1^+^ exchange at low pH on the observed exchange kinetics for ES2^+^, as observed from explicit kinetic simulations accounting for all three states, ES1^+^⇌GS⇌ES2^+^ (Supplementary Fig. S7B). In contrast to *k*_forward_, the reverse rate *k*_reverse_ = *k*_off_ ∼490 ± 40 s^−1^ was independent of pH (Fig. 7B), consistent with either the CS^prot^ (${{k}_{\textit{reverse}}} = {{k}_{\textit{prot}}} \times {{10}^{ - p{{K}_a}}}$) or IF^conf^ (${{k}_{\textit{reverse}}} = {{k}_{r,\textit{conf}}}$) mechanisms and proton-mediated proton transfer [74].

Since proton transfer is rate-limiting in CS^prot^ but not IF^conf^, the two pathways can in theory be distinguished using solvent KIEs [86, 87]. In CS^prot^ (and IF^prot^), the forward rate of the reaction depends on the diffusion-limited protonation rate. Thus, CS^prot^ predicts a significantly slower *k*_forward_ in 100% D_2_O versus H_2_O, typically with a *k*_H_/*k*_D_ ratio of ∼2–4 measured for various acids [86, 88]. The *k*_H_/*k*_D_ ratio for *k*_reverse_ is more difficult to predict because it depends on both the changes in the diffusion-limited protonation rate *k*_prot_ as well as the intrinsic p*K*_a_, which typically increases by ∼0.2–0.4 units in 100% D_2_O [88]. In contrast, IF^conf^ does not predict significant KIEs for either *k*_forward_ or *k*_reverse_, particularly if the intrinsic p*K*_a_ does not change significantly in 100% D_2_O.

A prior study measured GS⇌ES2 exchange in 100% D_2_O at pD = 6.4 [60]. As expected, the NMR-derived *p*_ES2_ ∼0.3% suggests a small increase in the apparent p*K*_a_ by ∼0.1 units in 100% D_2_O. Consistent with IF^conf^ but not CS^prot^, very small KIEs were measured for both *k*_on_ and *k*_off_ for the GS⇌ES2 exchange, with *k*_H_/*k*_D_ values of ∼0.8 ± 0.2 and ∼0.6 ± 0.1, respectively (Fig. 7C). The similar KIEs observed for GS⇌ES1 exchange, with *k*_H_/*k*_D_ values for *k*_on_ ∼1.4 and *k*_off_ ∼1.5, further support the IF^conf^ mechanism previously deduced based on modifications that shift the p*K*_a_ or alter the GS-ES1 conformational equilibrium [58].

It should be noted under certain circumstances, the IF^conf^ mechanism can also exhibit solvent KIEs despite protonation not being rate limiting, particularly if there are significant changes in the p*K*_a_ in 100% D_2_O leading to destabilization of the protonated intermediate. While such large changes in p*K*_a_ were not observed for ES1 and ES2, in cases where a significant change is observed in the p*K*_a_, additional evidence is needed to strongly establish the kinetic mechanism beyond just the solvent KIEs.

As an additional independent test, we analyzed the impact of mutations that alter the GS-ES2 conformational equilibrium in favor of ES2. In addition to TAR^ES2^, the G28U mutation also inverts the GS-ES2 conformational equilibrium [61], such that ES2 has an equilibrium population of ∼99.2% [61]. Because these mutations alter residues more than three bps away from the protonated C24–C39 mismatch, they can be safely assumed to minimally impact its intrinsic p*K*_a_. Assuming these mutations also do not alter the dominant kinetic pathway, CS^prot^ predicts an increase in *k*_forward_ = *f*_ES2_  *k*_prot_ [H^+^] by increasing *f*_ES2_ = *K*_conf_/(*K*_conf _+ 1), without affecting *k*_reverse_, and independent of the changes in *k*_ex,conf_ = *k*_f,conf_ + *k*_r,conf_. In contrast, IF^conf^ predicts that both *k*_forward_ and *k*_reverse_ can vary significantly, depending on how the mutation impacts the forward and reverse rates of the conformational change step (*k*_f,conf_ and *k*_r,conf_).

TAR^ES2^ and TAR^G28U^ alter $K_{\textit{conf}}^{ES2}$ by nearly three and five orders of magnitude, respectively. CS^prot^ predicts comparable changes to *k*_forward_ and no change to *k*_reverse_. Based on *R*_1ρ_ NMR measurements on TAR^ES2^ and TAR^G28U^, these mutations did not alter *k*_forward_ anywhere near the amount predicted by CS^prot^, increasing the rates only by one and three orders of magnitude, respectively (Fig. 7D). Moreover, they substantially decreased *k*_reverse_ by over two orders of magnitude, in poor agreement with CS^prot^ and in better agreement with IF^conf^ (Fig. 7D).

Finally, to ensure consistency of the pH-dependent *R*_1ρ_ measurements with the dominant IF^conf^ pathway, we performed a constrained three-state global fit assuming a linear topology (GS⇌GS^+^⇌ES2^+^) where the GS represents the combined GS + ES1^+^ conformations, which cannot be distinguished by the probes used for *R*_1ρ_ measurements. In our analysis, we assumed that the extra-helical C24 in the GS has a $pK_a^{GS}$ ∼4.0 similar to that deduced from NMR measurements on CTP [72]. We fixed the pH-dependent ES2^+^ population (*p*_ES2+_) according to the apparent $pK_a^{ES2}$ ∼4.0 deduced from a two-state fit of *R*_1ρ_ data_(1)_ and the pH-dependent GS^+^ population (*p*_GS+_) according to $pK_a^{GS}$ ∼4.0. The value of *k*_ex,prot_ was set to *k*_prot_([H^+^] + 10^−p^*^K^*^a,GS^) based on *k*_prot_ = 6 × 10^11^ M^−1^s^−1^ deduced for diffusion-limited protonation in nucleic acids from prior studies and the $pK_a^{GS}$ ∼4.0. Δω_GS+_, Δω_ES2+_, and *k*_ex,conf_ = *k*_f,conf _+ *k*_r,conf_ were allowed to vary during the fit.

With these constraints, we were able to satisfactorily fit the *R*_1ρ_ data for each of the three probes: U38-N3, U23-C6, and A35-C8, combining the five pHs (5.4, 6.0, 6.4, 7.0, and 7.4) with reduced χ^2^ ∼0.7–2.4 (Fig. 7E and Supplementary Fig. S7A), with the best-fit *k*_ex,conf_ from the three probes varying by <2-fold, in excellent agreement with each other. Consistent with conformational change being the rate-limiting step, the fits yielded an average *k*_ex,conf_ ∼693 ± 22 s^−1^, which was four orders of magnitude slower than the protonation step (*k*_ex,prot_ ∼4 × 10^7^ s^−1^). Furthermore, the best fit *k*_ex,conf_ was uniquely determined by our *R*_1ρ_ measurements, as increasing or decreasing the *k*_ex,conf_ led to a significantly worse fit (reduced χ^2^ > 10, Supplementary Fig. S7C). This analysis indicated that the forward and reverse rate constants of conformational change in the IF^conf^ pathway are 270 ± 9 s^−1^ and 423 ± 13 s^−1^, respectively (Fig. 7E). Because conformational change was rate-limiting and not protonation, reducing *k*_prot_ by 10-fold did not cause a significant deterioration in the agreement with the data (Supplementary Fig. S7C). The fitted Δω values for GS^+^ and ES2^+^ were also consistent with U38-N3, U23-C6, and A35-C8 primarily sensing conformational change (Supplementary Table S4), with U23-C6 showing deviations from the expected Δω values for GS^+^, likely due to overfitting to the U23-C6 *R*_1ρ_ profiles, which consistently have a lower signal-to-noise compared to U38-N3 and A35-C8.

The above IF^conf^ mechanism predicts that the protonated intermediate in which the bulge C24 is protonated should have a population of ∼7–16% at pH = 5.4. We tested this prediction by looking for chemical shift perturbations in TAR at C24-C5 and C24-C6, which should be sensitive to protonation of the cytosine bulge when lowering the pH from 6.4 to 5.4. Indeed, we observed a downfield shift for C24-C6 by ∼0.1 ppm and an upfield shift for C24-C5 by ∼0.8 ppm, which, assuming chemical shift perturbations of ∼1 ppm for C5 and ∼1.6 ppm for C6 upon protonation, yields an average population of ∼28% for the protonated intermediate, in good agreement with predictions (Supplementary Fig. S8). In contrast to C24, C39-C5, and C6 exhibit minimal chemical shift perturbation when lowering pH, consistent with C39 being base-paired with G26 in a Watson–Crick geometry, where its N3 atom hydrogen bonds with the G26 imino proton and is shielded from protonation.

## Discussion

Some of the earliest studies of micro-to-millisecond timescale motions in RNA revealed pH-dependent dynamics tightly coupled to protonation of nucleobases involved in mismatches [18, 89]. Since then, several studies have characterized sparsely populated RNA conformational states that are protonation-coupled typically through protonated C–A^+^ and G–A^+^ mismatches [5, 14, 23, 26, 46, 90]. TAR ES2 joins this growing list of sparsely protonated RNA conformational states this time specifically through a C^+^–C mismatch. Because the NMR chemical shift signatures of cytosine protonation are more subtle relative to the very large chemical shift changes accompanying protonation of adenine-N1, its detection proved more difficult in TAR ES2. This motivates development of NMR experiments that can detect cytosine-N3 and that are likely to be very sensitive to protonation of this base, as shown previously for adenine-N1 protonation [91], along with orthogonal NMR measurements, including proton chemical exchange and scalar couplings, to characterize the specific interactions in the protonated C^+^–C mismatch in ES2. Our findings also underscore the importance of performing NMR dynamics measurements across a broad pH range when determining whether a rare RNA conformational state is protonated, especially when the equilibrium population of the transient state is low, as this can depress the overall apparent p*K*_a_ to values below the range typically explored experimentally.

**Figure 8.**
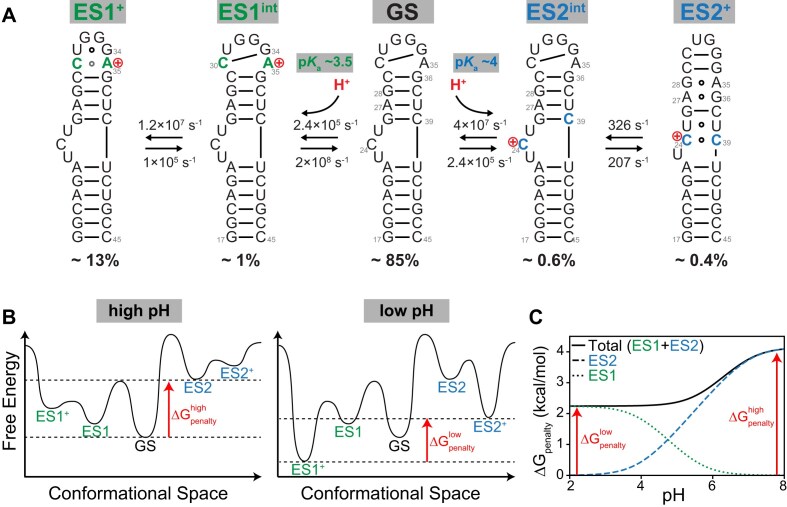
pH-dependent competition between two TAR protonated excited conformational states. (**A**) Secondary structural depictions of the kinetic mechanisms for proton-coupled conformational transitions in HIV-1 TAR involving ES1 (left) and ES2 (right). Both ESs are stabilized by protonation of A35 in ES1 and C24 in ES2 via the induced-fit pathway, leading to formation of protonated C30-A^+^35 and C^+^24-C39 mismatches in ES1^+^ and ES2^+^, respectively. (**B**) Shown are representative depictions of the pH-dependent free energy landscape for TAR, comprising GS, ES1, and ES2, with ES1 and ES2 both forming protonated alternative conformational states (ES1^+^, ES2^+^). At high pH, both excited states predominantly exist in their neutral form; hence, the conformational penalty to adopt ES2 is primarily determined by the energetic difference between the dominant GS versus ES2. Interestingly, at low pH, while the protonation of ES2 decreases this energetic penalty continuously, the protonation of ES1 instead increases the penalty, thereby leading to combined competitive effect on the pH-dependent conformational penalty to adopt ES2, specifically at low pH, where the penalty to adopt ES2 is primarily determined by the energetic difference between the dominant ES1^+^ versus ES2^+^. (**C**) Shown are the total predicted contributions to the conformational penalty to adopt ES2 in TAR (solid line) based on the best-fit thermodynamic model for pH between 2 and 8. Also shown are the individual contributions from the pH-dependent stabilization of ES2 (dashed line) and ES1 (dotted line).

We recently elucidated the kinetic mechanism underlying protonation of TAR ES1 and showed that it proceeds via an induced-fit mechanism in which the solvent-exposed A35 nucleobase is rapidly protonated followed by a slower conformational change that zips up the apical loop and results in the C–A^+^ mismatch [58] (Fig. 8A). Despite the involvement of a different protonated C^+^–C mismatch and a larger-scale rearrangement in secondary structure, which is significantly slower than that of ES1, our results show that TAR ES2 also forms by a similar induced-fit mechanism whereby the solvent-exposed C24 bulge is rapidly protonated followed by the conformational change to form the C^+^24-C39 mismatch (Fig. 8A). Such an induced-fit pathway was previously proposed for the proton-coupled nucleobase-flipping of a uridine residue in U6-RNA intramolecular stem loop, where rapid protonation of an adenine residue occurred first to form a protonated C–A^+^ mismatch, followed by slower (over 10-fold) conformational change involving flipping of the uridine residue inside the helix [18]. The fact that the same induced-fit mechanism was observed for ES1 and ES2 across different protonated mismatches and despite the *k*_on_ = (8.2 ± 3.4) x 10^6^ M^−1^s^−1^ for GS⇌ES2 being nearly three orders of magnitude slower than *k*_on_ ∼10^9^ M^−1^ s^−1^ for GS⇌ES1 due to its much slower conformational rearrangement points to the robustness of this kinetic mechanism in cases where the protonated residues are flipped out and solvent accessible in the GS.

In sharp contrast, distinct mechanisms can arise when the protonated residues are not solvent exposed in the GS. In particular, we previously showed that the transition between Watson–Crick and protonated G*_syn_*-C^+^ Hoogsteen bps in duplex DNA proceeds via a conformational selection mechanism involving rate-limiting protonation of a lowly populated neutral G*_syn_*-C Hoogsteen intermediate. In this case, and in contrast to TAR ES1, ES2, as well as the U6-RNA, the cytosine-N3 protonation site is occluded in the canonical Watson–Crick GS due to hydrogen bonding, ruling out an induced-fit mechanism.

It is possible that an induced-fit pathway in which conformational change is rate-limiting is generally favored when the protonated nucleobases are solvent accessible in the GS, given the rapid rate of diffusion-limited protonation and given that conformational transitions involving substantial changes in secondary structures, which require breaking multiple bp, tend to be slow. Interestingly, for most protonated ESs reported to date, including HIV-1 RRE stem IIB [7] and the precursor of microRNA-21 [5], the nucleobase that is protonated in the mismatch was also solvent-accessible in either a bulge or internal loop motif in the GS. Thus, a similar induced-fit mechanism may be at play in these RNAs as well. Future studies should dissect the consequence of these varying kinetic pathways on cellular function.

Our results reveal how competition between two protonated conformations creates a complex, non-monotonic pH-dependent ensemble, which cannot be captured by conventional two-state models (Fig. 8A). Consider, for example the thermodynamic energetic penalty associated with forming ES2, given by $\Delta {{G}_{\textit{penalty}}} = - RT\ln ( {{{p}_{ES2}} + {{p}_{ES2 + }}} ),\ $which could be incurred by any biochemical process acting on ES2 (e.g. binding of a small molecule therapeutic). In the absence of ES1, lowering the pH would lead to systematic stabilization of the protonated ES2^+^, with its population asymptotically reaching ∼100%. This corresponds to a monotonous decrease in $\Delta {{G}_{\textit{penalty}}}$ upon lowering pH, which asymptotically reaches $\Delta {{G}_{\textit{penalty}}}$ ∼0 at low pH. In contrast, upon increasing the pH, the neutral ES2 conformation dominates, and the associated $\Delta {{G}_{\textit{penalty}}}$ increases to a maximum value with increasing pH of $\Delta {{G}_{\textit{penalty}}}$ ~4.1 kcal/mol (Fig. 8B).

However, the pH-dependent energetics are quite different in the presence of a competing protonated ES1^+^. The pH-dependent stabilization of ES1 (p*K*_a_ ∼7.5) competes against ES2^+^ when lowering pH, increasing the $\Delta {{G}_{\textit{penalty}}}$ penalty to form ES2^+^ compared to the case in which ES1^+^ did not exist (Fig. 8B). For example, the penalty increases from <0.5 kcal/mol without ES1 to ∼2.2 kcal/mol with ES1 at pH < 5.4. The effective $\Delta {{G}_{\textit{penalty}}}$ observed experimentally arises from the sum of these two competing contributions, resulting in the lowest energetic penalty of forming ES2 of ∼2.2 kcal/mol, even at very low pH (Fig. 8C). Similar considerations likely extend to the kinetics of transition between states, where competition between protonated species may influence rate-limiting steps and transition pathways. Future studies should focus on characterizing such proton-coupled conformational transitions in various RNAs, including in the presence of varying concentrations of monovalent and divalent cations [81, 92, 93], to further dissect their role in modulating RNA cellular function. In particular, our approach can be used to examine how changing the type and concentration of metal cations [67, 94, 95] impacts such exchange involving rare protonated conformational states.

While our RD measurements suggest that C24 is likely the site of protonation, we cannot rule out the possibility that the protonated C–C mismatch in ES2 exists in a rapid dynamic equilibrium between the C^+^24-C39 and C24-C^+^39 states, as also suggested by the FARFAR-NMR ensemble for TAR^ES2^, which showed equal populations of the C^+^24-C39 and C24-C^+^39 wobble geometries. The preference for protonating C24 versus C39 will be dictated by the apparent pK_a_ values of the two cytosine bases, which in turn will depend on stacking interactions with immediate neighbors and cation-pi interactions, whose sequence dependence remains poorly characterized. Based on the FARFAR-NMR TAR^ES2^ ensemble, the U25-U38 mismatch neighboring C24–C39 adopts two alternative wobble conformations, U25(C4)-U38(C2) and U25(C2)-U38(C4), in an ~55:45 equilibrium [64]. U25(C2)-U38(C4) appears to stack more optimally with the neighboring C24–C39, while U25(C4)-U38(C2) forms a broader sub-ensemble with diminished stacking interactions with C24–C39, and the junctional A22-U40 is only partially formed. Within the ensemble, C^+^24-C39 and C24-C^+^39 wobbles are populated at similar levels. Notably, when C24–C39 adopts the C^+^24-C39 wobble, the neighboring U25-U38 adopts the U25(C2)-U38(C4) wobble; conversely, when C24–C39 forms the C24-C^+^39 wobble, U25-U38 adopts the U25(C4)-U38(C2) wobble. Overall, stacking with neighbors is more optimal in the C^+^24-C39 conformation, which we assigned as the major protonated state. These coupled conformational preferences between adjacent mismatches, likely mediated by stacking interactions, highlight potential cooperative behavior within the protonated ensemble that warrants deeper investigation in future work.

While no functional role has been assigned to the ES2 conformational transition in the TAR ensemble, its inhibition of transcriptional activity suggests that ES2 could act as a molecular switch by facilitating the release of Tat:SEC from the complex, thereby enabling multiple rounds of transcriptional activation. In addition, prior studies demonstrate that mutations stabilizing ES2 promote TAR–TAR kissing-loop dimerization, which is proposed to play roles during genome dimerization and packaging [96, 97]. A recent study characterizing the impact of mutations on HIV genome dimerization revealed that ES2-stabilizing point substitution mutations such as G28C, G28U, and G36U significantly enhanced dimerization, whereas GS-retaining mutations such as A22G, U40C, and U31C had little effect on dimerization [98]. These observations highlight the potential importance of such conformational transitions for biological activity of HIV-1 TAR and the need to further dissect their specific roles in cellular function.

By enabling precise dissection of protonation sites, p*K*_a_ values, and kinetic mechanisms for multiple and competing protonated species, the NMR methods presented here promise to provide critical insights into how RNA ensembles respond to changes in pH, shedding light on RNA behavior in diverse physiological contexts and informing efforts to engineer RNA for therapeutic and synthetic applications.

## Supplementary Material

gkaf1366_Supplemental_File

## Data Availability

All scripts for data analysis and plotting, kinetic simulations, and three-state NMR RD fits used in this manuscript are available at GitHub: 10.5281/zenodo.15599158.

## Notes

Present address: Yusuf Hamied Department of Chemistry, University of Cambridge, Cambridge, UK

Present address: MRC Laboratory of Molecular Biology, Cambridge, UK
