# Supplementary material for: Revealing hidden protonated conformational states in RNA dynamic ensembles
Source: Nucleic Acids Res. 2025 Dec 18;53(22):gkaf1366. doi: 10.1093/nar/gkaf1366 (PMC12714568; doi:10.1093/nar/gkaf1366)
Supplement: gkaf1366_Supplemental_File [file gkaf1366_supplemental_file.pdf]

**Supporting Information**

# Revealing hidden protonated conformational states in RNA dynamic ensembles

Ainan Geng[1‡], Rohit Roy[2‡], Laura Ganser[1], Linshu Li[3], Hashim M. Al-Hashimi[3*]

[‡]These two authors contributed equally to this work.

[1]Department of Biochemistry, Duke University School of Medicine, Durham, NC 27710, USA

[2]Center for Genomic and Computational Biology, Duke University School of Medicine, Durham, NC 27710, USA

[3]Department of Biochemistry and Molecular Biophysics, Columbia University, New York, New York 10032, United States.

* To whom correspondence should be addressed.

Email: ha2639@cumc.columbia.edu

## Supplementary Figures

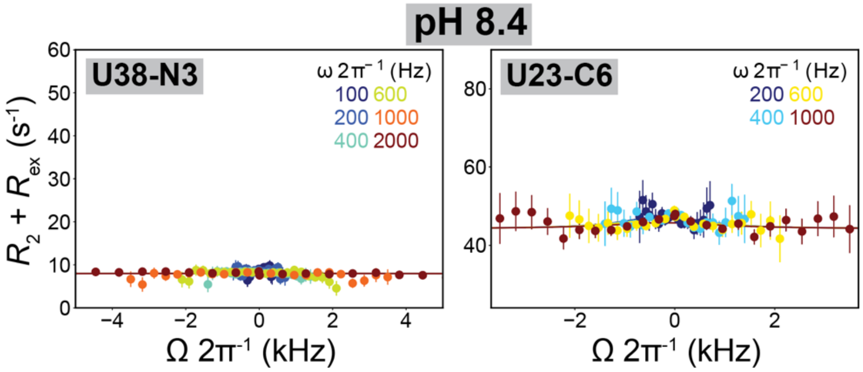


**Supplementary Figure S1. Off-resonance $R_{1\rho}$ profile for ES2 in TAR at pH 8.4.** Off-resonance $^{15}N$ and $^{13}C$ $R_{1\rho}$ relaxation dispersion profiles measured for U38-N3 and U23-C6 at pH = 8.4. Spin-lock powers are color-coded. Solid lines represent the global 2-state fits using the Bloch–McConnell equation (see Methods). Error bars indicate ±1 standard deviation from Monte Carlo simulations (500 iterations per measurement). All NMR experiments were conducted in buffer containing 15 mM sodium phosphate, 25 mM NaCl, and 0.1 mM EDTA. The experimental data were collected on a 900 MHz spectrometer.

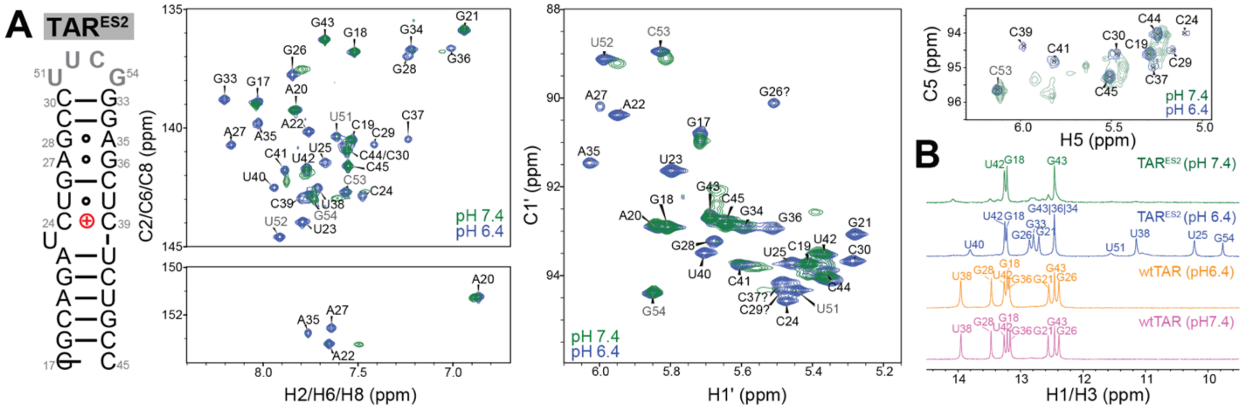


**Supplementary Figure S2. Exchange broadening of resonances in TAR$^{ES2}$ under high pH. (A)** The secondary structure (left) and overlays (right) of 2D $^{1}$H–$^{13}$C HSQC spectra of TAR$^{ES2}$ at pH 7.4 (in green) on the corresponding spectra of TAR$^{ES2}$ at pH 6.4 (in green) for the aromatic (C8/6/2-H8/6/2), sugar (C1′H1′), and C5H5 resonances. Note, the UUCG tetraloop residues are labelled from 51 to 54. **(B)** Overlay of 1D $^{1}$H imino (H1/H3) spectra measured for wtTAR and TAR$^{ES2}$ at pH 6.4 and 7.4, showing that only TAR$^{ES2}$ exhibits strong pH-dependent exchange broadening upon increasing the pH from 6.4 to 7.4.

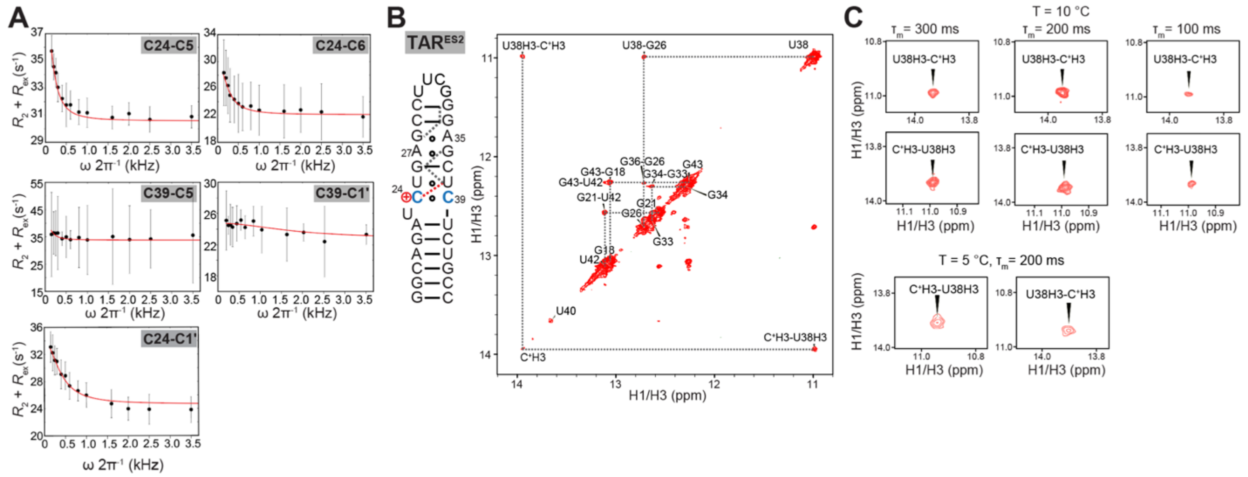


**Supplementary Figure S3. Protonation of the C24–C39 mismatch. (A)** The $^{13}$C on-resonance $R_{1\rho}$ profiles measured for C39 and C24 in wtTAR at pH = 5.4. All NMR experiments were conducted in buffer containing 15 mM sodium phosphate, 25 mM NaCl, and 0.1 mM EDTA. The experimental data were collected on a 600 MHz spectrometer. **(B)** Shown is the imino region of the 2D $^{1}$H-$^{1}$H NOESY spectra for TAR$^{ES2}$ at pH = 6.4, highlighting potential imino-imino NOE cross peaks suggesting the formation of a protonated C$^{+}$24-C39 in TAR$^{ES2}$. The 2D NOESY spectrum was recorded on a 900 MHz spectrometer at 5 ºC in 10% $D_2O$ with a mixing time ($\tau_m$) of 200 ms. **(C)** The imino NOE cross peaks observed in the 2D $^{1}$H-$^{1}$H NOESY spectra between the C$^{+}$24-C39 mismatch and the U25-U38 mismatch in TAR$^{ES2}$ at varying temperatures (T= 10 and 5 ºC) and mixing times ($\tau_m$ = 300, 200 and 100 ms).

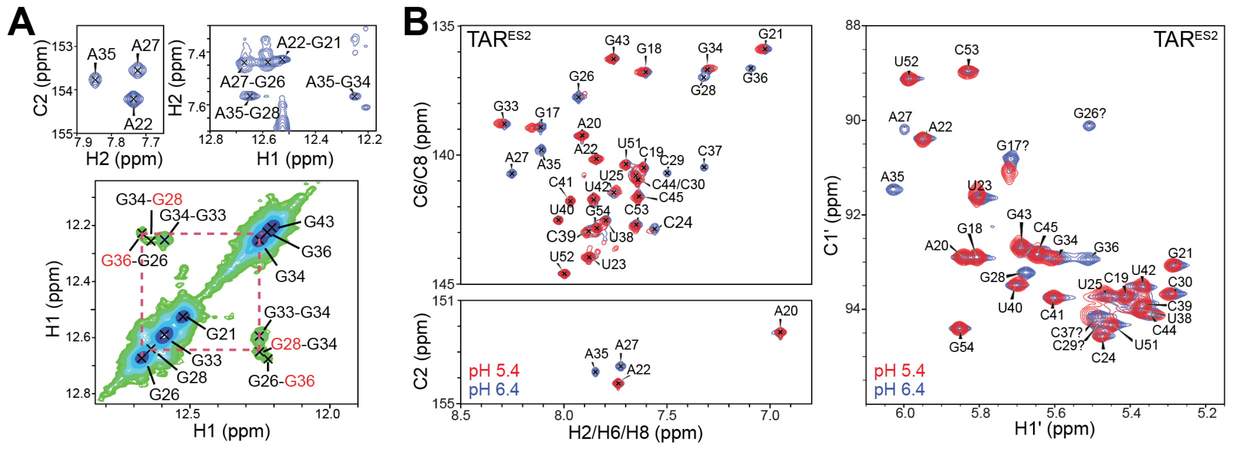

**Supplementary Figure S4. Lowering the pH to 5.4 protonates the G-A mismatch. (A)** Shown on left is the 2D $^{1}H$–$^{13}C$ HSQC spectra for the aromatic C2H2 resonances in TAR$^{ES2}$ at pH 6.4, highlighting lack of exchange broadening of the C2H2 resonances of either A35 or A27, which would be expected if the A-G mismatches in TAR$^{ES2}$ were protonated. Also shown (on right and bottom) are regions of the 2D $^{1}H$-$^{1}H$ NOESY spectra for TAR$^{ES2}$ at pH = 6.4, highlighting critical H2-H1 and H1-H1 NOE cross peaks which demonstrate that G28 and G36 adopts the *anti* and not the *syn* conformation in TAR$^{ES2}$. The HSQC spectrum was recorded at 25 °C, while the NOESY spectrum was recorded at 5 °C with a mixing time ($\tau_m$) of 200 ms. **(B)** Overlay of 2D $^{1}H$–$^{13}C$ HSQC spectra of TAR$^{ES2}$ at pH 5.4 (in red) on the corresponding spectra of TAR$^{ES2}$ at pH 6.4 (in blue) for the aromatic (C8/6/2-H8/6/2) and sugar (C1′H1′).

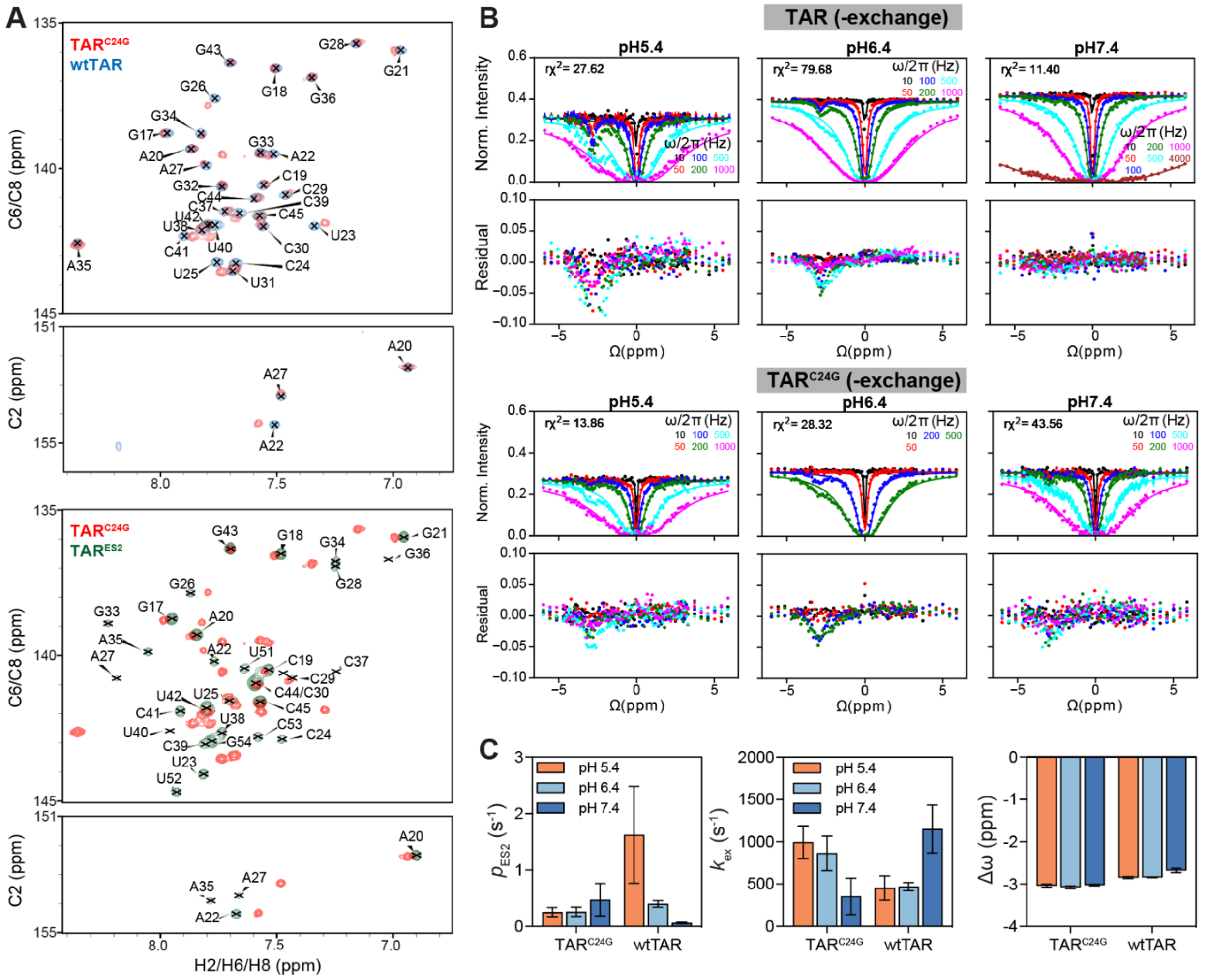

**Supplementary Figure S5. TAR$^{C24G}$ decouples protonation from conformational exchange. (A)** TAR$^{C24G}$ mutant adopts a GS-like conformation. Shown are overlays of 2D $^{1}$H–$^{13}$C HSQC spectra of TAR$^{C24G}$ at pH 6.4 (in red) on the corresponding spectra of wtTAR at pH 6.4 (blue, top) and TAR$^{ES2}$ at pH 6.4 (green, bottom) for the aromatic (C8/6/2-H8/6/2). **(B)** pH-dependent $^{1}$H CEST profiles measured for U38-H3 in TAR (top) and the TAR$^{C24G}$ mutant (bottom). The different RF powers applied are color-coded. All NMR experiments were conducted in buffer containing 15 mM sodium phosphate, 25 mM NaCl, and 0.1 mM EDTA. A fit of the $^{1}$H CEST profiles assuming no exchange contribution is shown as solid lines. Residual plots (measured normalized intensity – fit normalized intensity) are shown below each CEST profile. Error bars (often smaller than the data points) represent the standard deviation from triplicate CEST measurements of the peak intensity using zero relaxation delay collected at each RF field. **(C)** Bar plots depicting the variations in best-fit exchange parameters $p_{ES2}$, $k_{ex}$, and $\Delta\omega$ obtained from fitting the $^{1}$H CEST profiles assuming 2-state exchange at varying pH = 5.4, 6.4, and 7.4 for wtTAR and TAR$^{C24G}$.

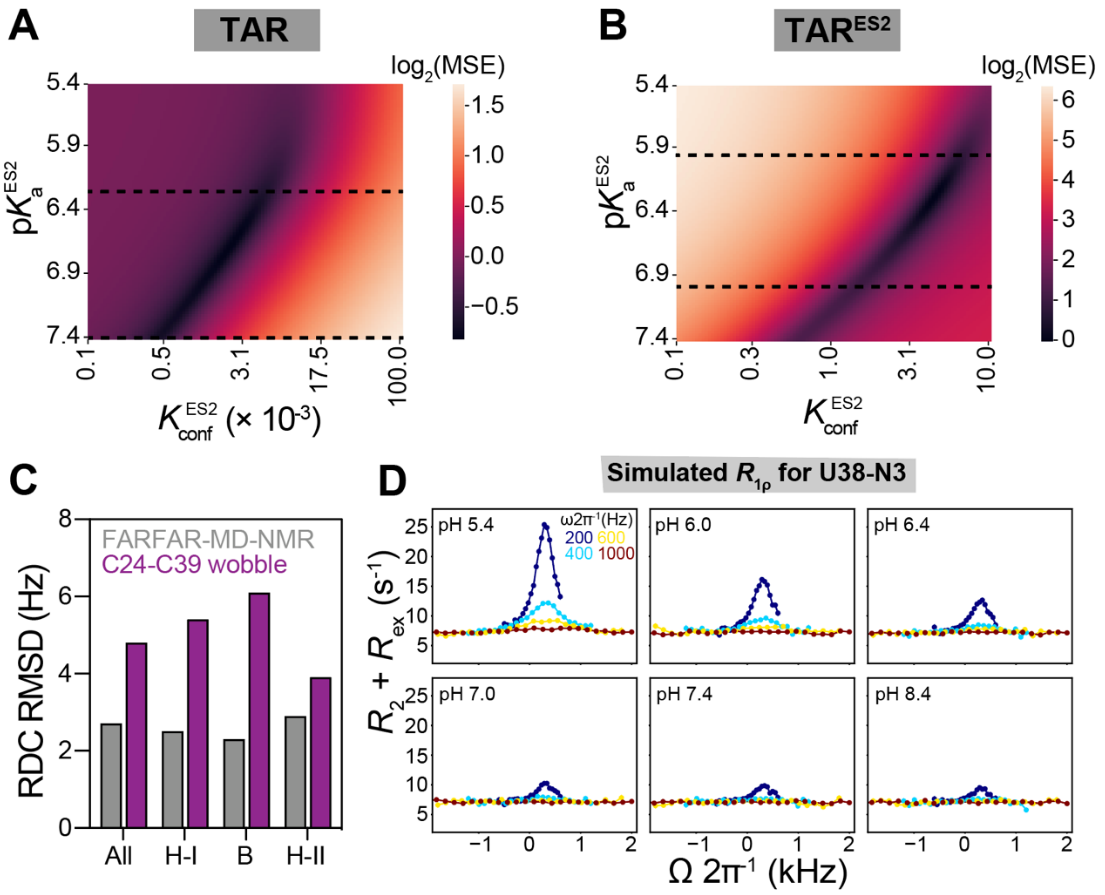


**Supplementary Figure S6. Testing the five-state thermodynamic model obtained from pH dependent ES2 populations. (A-B)** Degeneracy test of the thermodynamic parameters for **(A)** TAR and **(B)** TAR$^{ES2}$. Shown are the heatmaps of the mean squared error (MSE, $\log_2$ scale) between the $p_{ES2}$ measured from NMR and predicted by the corresponding thermodynamic models obtained by systematically varying $pK_a^{ES2}$ and $K_{conf}^{ES2}$. For wtTAR, $pK_a^{ES2}$ was varied from 5.4 to 7.4, and $K_{conf}^{ES2}$ from $10^{-4}$ to $10^{-1}$. For TAR$^{ES2}$, $pK_a^{ES2}$ was varied from 5.4 to 7.4, and $K_{conf}^{ES2}$ from $10^{-1}$ to 10. Black dashed lines identify the range of values for $pK_a^{ES2}$which produce a good fit to the data with MSE within ~2-fold of the best-fit model. Based on the heatmaps, we can conclude that the intrinsic $pK_a^{ES2}$ is > 6.4. **(C)** The measured versus predicted RDC RMSDs for the best-fit ensembles (Methods) comprising all different protonated and neutral conformations (Supplementary Figure S3A, grey) vs that containing only the protonation competent C24-C39 wobble conformations (magenta). Constraining C24-C39 to a protonated wobble conformation significantly increases RDC RMSD across all motifs, including lower helix (H-I), bulge (B), and upper stem (H-II). **(D)** Shown are simulated off-resonance $^{15}$N $R_{1\rho}$ profiles for U38-N3 in TAR depicting the exchange between the GS and ES2 at varying pH from 5.4-8.4. We used the best fit intrinsic $pK_a^{ES2}$ ~7.1 ± 0.5 and $K_{conf}^{ES2}$ ~0.9 x $10^{-3}$ to predict the population of both neutral ES2 and protonated ES2$^{+}$ as a function of pH. We assumed that the protonated and neutral ES2 conformations exists in rapid exchange on the chemical shift timescales to estimate the total ES2 population ($p_{ES2,Total}$ = $p_{ES2}$ + $p_{ES2+}$) comprising both protonated and neutral species, and used the

pH-dependence of the forward and reverse rates of exchange to extrapolate the $k_{ex}$ across the varying pH conditions. The value of $k_{ex}$ was deduced based on $k_{ex} = k_{on} [H^+] + k_{off}$ in which $k_{on}$ ~8.2 x $10^6$ $M^{-1}s^{-1}$ and $k_{off}$ ~490 $s^{-1}$ based on a 2-state global fit of the $R_{1\rho}$ data at pH = 5.4 – 7.4. Finally, using the above described $p_{ES2,Total}$ and $k_{ex}$, we simulated the $R_{1\rho}$ profiles for U38-N3 at varying pH from 5.4-8.4, assuming a random simulated error of ~2% to accurately mimic NMR experimental conditions.

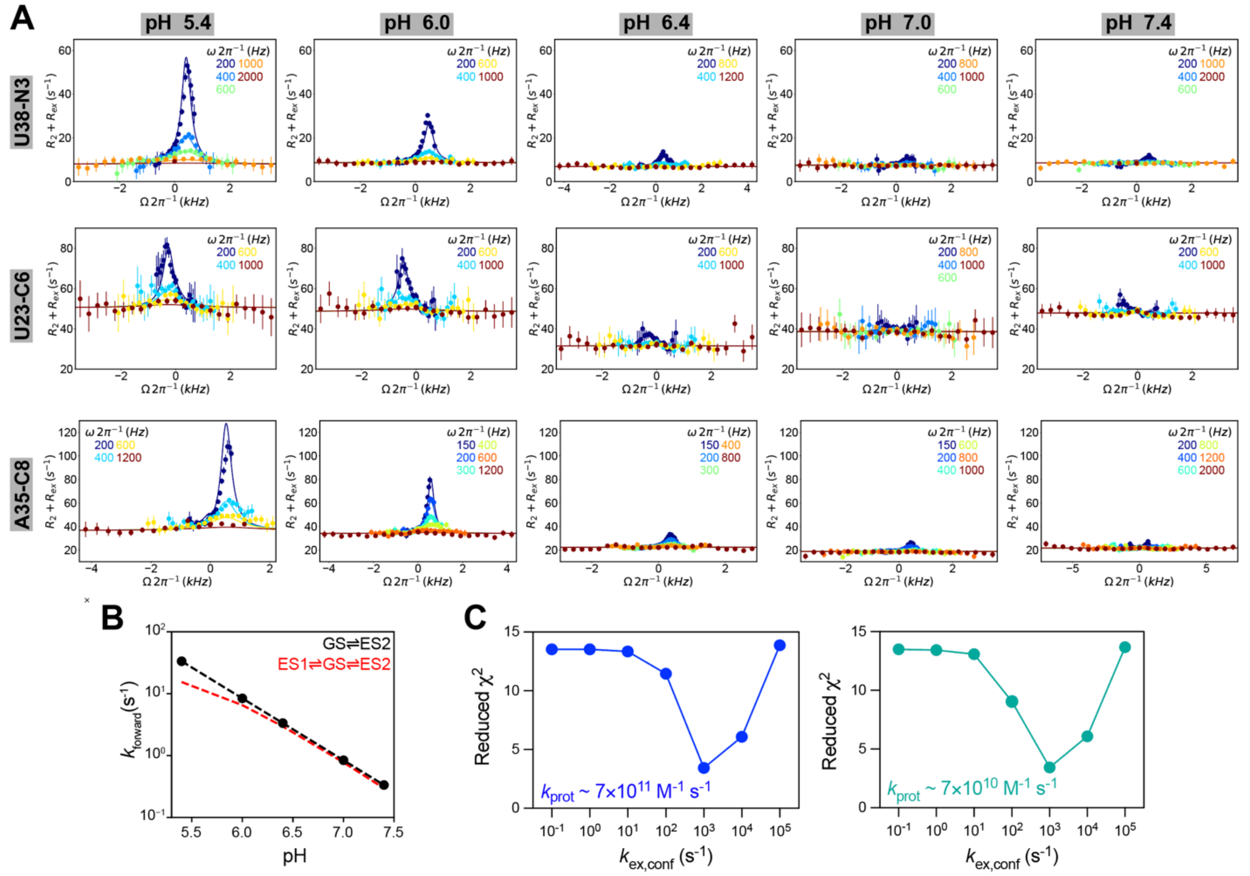


**Supplementary Figure S7. Three-state fits of NMR off-resonance $R_{1\rho}$ profiles at variable pH.** **(A)** Shown are the off-resonance $^{13}$C $R_{1\rho}$ profiles measured for TAR depicting the ES2 exchange for probes U38-N3, U23-C6 and A35-C8 at pH = 5.4, 6.0, 6.4, 7.0 and 7.4. Spin-lock powers are color coded. Solid lines denote fits to the data using the Bloch–McConnell equations assuming the 3-state IF model, as described in Methods. Error bars represent the experimental uncertainty in the measured $R_{1\rho}$ data and were computed as ± 1 s.d. from Monte Carlo simulations (number of iterations = 500) for one measurement as previously described.[2] **(B)** Comparison of the pH-dependence of $k_{forward}$ for the formation of ES2 from (i) simplified 2-state model GS⇌ES2$^+$ (in black) and a 3-state model incorporating both protonated excited states ES1$^+$⇌GS⇌ES2$^+$ (in red) showing the predicted deviations at pH = 5.4-7.4 obtained from explicit kinetic simulations (see methods). **(C)** The reduced $\chi^2$ obtained from a global 3-state fit of the U38-N3 $R_{1\rho}$ data calculated as a function of varying $k_{ex,conf}$ = $10^{-1}$ – $10^5$ $s^{-1}$ and $k_{prot}$ = 6 x $10^{11}$ $M^{-1}$ $s^{-1}$ (left, blue) and 6 x $10^{10}$ $M^{-1}$ $s^{-1}$ (right, cyan).

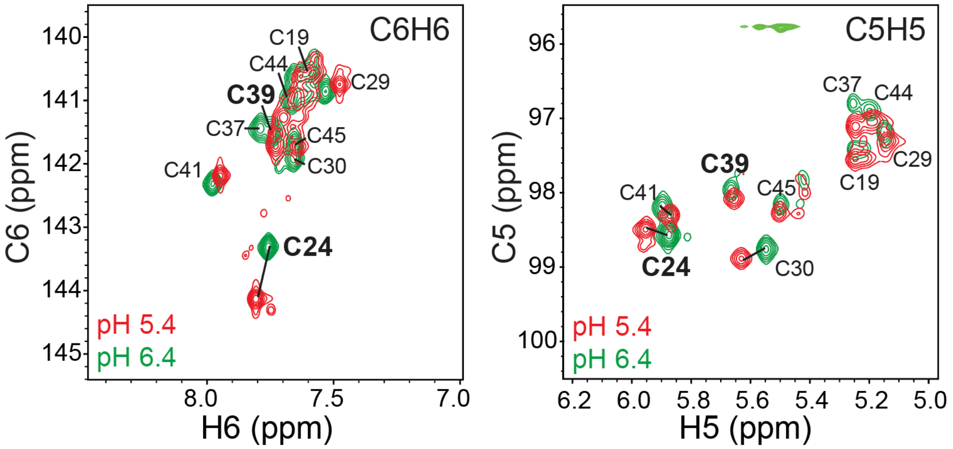


**Supplementary Figure S8. pH-dependent changes in chemical shifts provide evidence for the protonated intermediate in the IF$^{conf}$ pathway**. Shown is the overlay of 2D $^{1}$H–$^{13}$C HSQC spectra of TAR at pH 5.4 (in red) on the corresponding spectra of TAR at pH 6.4 (in green) for the aromatic (C8/6/2-H8/6/2) and C5H5 resonances, both recorded at 25 °C.

## Supplementary Tables

**Supplementary Table S1.** Summary of exchange parameters obtained from a 2-state global fit of the TAR $R_{1\rho}$ data detecting the ES2 population measured at pH = 5.4, 6.0, 6.4, 7.0 and 7.4. Errors represent the experimental uncertainty in the measured $R_{1\rho}$ data and were computed as ± 1 s.d. from Monte Carlo simulations (number of iterations = 500) for one measurement as previously described.[2]

| **pH = 5.4, T = 25°C, 10% D2O** | | | |
|---|---|---|---|
| Parameter | C24-C5 | C24-C6 | C24-C1′ |
| $p_{minor}$ (%) | 1.50 ± 0.10 | | |
| $k_{ex}$ ($s^{-1}$) | 618 ± 58 | | |
| Δω (ppm) | -1.47 ± 0.05 | -1.26 ± 0.03 | 2.54 ± 0.05 |
| $R_1$ ($s^{-1}$) | 2.59 ± 0.13 | 2.60 ± 0.08 | 1.97 ± 0.09 |
| $R_2$ ($s^{-1}$) | 21.97 ± 0.17 | 30.45 ± 0.11 | 24.64 ± 0.15 |
| Reduced $\chi^2$ | 0.49 | | |
| **pH = 5.4, T = 25°C, 10% $D_2O$** | | | |
| Parameter | U38-N3 | U23-C6 | A35-C8 |
| $p_{minor}$ (%) | 1.70 ± 0.17 | | |
| $k_{ex}$ ($s^{-1}$) | 562 ± 70 | | |
| Δω (ppm) | -5.14 ± 0.06 | 1.70 ± 0.05 | -2.68 ± 0.03 |
| $R_1$ ($s^{-1}$) | 1.6 ± 0.05 | 1.68 ± 0.11 | 1.07 ± 0.08 |
| $R_2$ ($s^{-1}$) | 8.8 ± 0.13 | 52.54 ± 0.29 | 39.31 ± 0.22 |
| Reduced $\chi^2$ | 0.64 | | |
| **pH = 6.0, T = 25°C, 10% $D_2O$** | | | |
| $p_{minor}$ (%) | 1.01 ± 0.14 | | |
| $k_{ex}$ ($s^{-1}$) | 408 ± 67 | | |
| Δω (ppm) | -5.11 ± 0.05 | 2.15 ± 0.08 | -2.54 ± 0.02 |
| $R_1$ ($s^{-1}$) | 1.26 ± 0.02 | 1.79 ± 0.09 | 1.36 ± 0.03 |
| $R_2$ ($s^{-1}$) | 8.19 ± 0.06 | 49.39 ± 0.26 | 33.81 ± 0.1 |
| Reduced $\chi^2$ | 0.67 | | |
| **pH = 6.4, T = 25°C, 10% $D_2O$ from prior study[2]** | | | |
| $p_{minor}$ (%) | 0.40 ± 0.05 | | |
| $k_{ex}$ ($s^{-1}$) | 474 ± 69 | | |
| Δω (ppm) | -5.2 ± 0.2 | 2.3 ± 0.1 | -2.4 ± 0.04 |
| $R_1$ ($s^{-1}$) | 1.4 ± 0.03 | 2.5 ± 0.04 | 2.13 ± 0.02 |
| $R_2$ ($s^{-1}$) | 6.2 ± 0.03 | 30.7 ± 0.1 | 18.5 ± 0.04 |

| | | | |
|---|---|---|---|
| Reduced $\chi^2$ | | | |
| **pH = 7.0, T = 25°C, 10% $D_2O$** | | | |
| $p_{minor}$ (%) | 0.17 ± 0.01 | | |
| $k_{ex}$ ($s^{-1}$) | 877 ± 113 | | |
| Δω (ppm) | -4.7 ± 0.2 | 1.8 ± 0.2 | -2.3 ± 0.1 |
| $R_1$ ($s^{-1}$) | 1.03 ± 0.02 | 2.4 ± 0.06 | 1.9 ± 0.02 |
| $R_2$ ($s^{-1}$) | 7.1 ± 0.04 | 38.39 ± 0.16 | 18.18 ± 0.05 |
| Reduced $\chi^2$ | 0.34 | | |
| **pH = 7.4, T = 25°C, 10% $D_2O$** | | | |
| $p_{minor}$ (%) | 0.50 ± 0.37 | | |
| $k_{ex}$ ($s^{-1}$) | 123 ± 98 | | |
| Δω (ppm) | -4.8 ± 0.3 | 2.5 ± 0.3 | -2.3 ± 0.2 |
| $R_1$ ($s^{-1}$) | 1.47 ± 0.02 | 1.92 ± 0.05 | 1.5 ± 0.03 |
| $R_2$ ($s^{-1}$) | 8.3 ± 0.04 | 47.58 ± 0.14 | 21.43 ± 0.05 |
| Reduced $\chi^2$ | 0.99 | | |

**Supplementary Table S2.** Summary of exchange parameters obtained from a 2-state global fit of the TAR$^{ES2}$ $R_{1\rho}$ data detecting the GS-like population measured at pH = 5.4, 6.0 and 6.4. Errors represent the experimental uncertainty in the measured $R_{1\rho}$ data and were computed as ± 1 s.d. from Monte Carlo simulations (number of iterations = 500) for one measurement as previously described.[2]

| **pH = 5.4, T = 25°C, 10% $D_2O$** | | |
|---|---|---|
| Parameter | U38-N3 | U23-C6 |
| $p_{minor}$ (%) | 5.56 ± 1.13 | |
| $k_{ex}$ ($s^{-1}$) | 110 ± 25 | |
| Δω (ppm) | 5.1 ± 0.1 | -1.8 ± 0.1 |
| $R_1$ ($s^{-1}$) | 1.63 ± 0.04 | 2.07 ± 0.08 |
| $R_2$ ($s^{-1}$) | 8.56 ± 0.11 | 32.73 ± 0.21 |
| Reduced $\chi^2$ | 1.47 | |
| **pH = 6.0, T = 25°C, 10% $D_2O$** | | |
| $p_{minor}$ (%) | 11.29 ± 0.74 | |
| $k_{ex}$ ($s^{-1}$) | 90 ± 7 | |
| Δω (ppm) | 5.3 ± 0.03 | -2.3 ± 0.02 |
| $R_1$ ($s^{-1}$) | 2.14 ± 0.04 | 2.27 ± 0.06 |
| $R_2$ ($s^{-1}$) | 9.41 ± 0.09 | 33.14 ± 0.17 |
| Reduced $\chi^2$ | 0.52 | |
| **pH = 6.4, T = 25°C, 10% $D_2O$** | | |
| $p_{minor}$ (%) | 12.62 ± 1.28 | |
| $k_{ex}$ ($s^{-1}$) | 121 ± 15 | |
| Δω (ppm) | 5.3 ± 0.03 | -2.4 ± 0.04 |
| $R_1$ ($s^{-1}$) | 2.63 ± 0.04 | 2.04 ± 0.2 |
| $R_2$ ($s^{-1}$) | 10.25 ± 0.14 | 37.55 ± 0.46 |
| Reduced $\chi^2$ | 95.12 | |

**Supplementary Table S3.** Summary of exchange parameters obtained from a 2-state global fit of the wtTAR and $TAR^{C24G}$ $^1H$ CEST data detecting the ES2 population measured at pH = 5.4, 6.4 and 7.4. Errors represent the experimental uncertainty in the measured $R_{1\rho}$ data and were computed as ± 1 s.d. from Monte Carlo simulations (number of iterations = 500) for one measurement as previously described.[2]

| **Param** | **wtTAR** | | | **C24G** | | |
|---|---|---|---|---|---|---|
| **pH** | **5.4** | **6.4** | **7.4** | **5.4** | **6.4** | **7.4** |
| $p_{ES}$ (%) | 1.62±0.86 | 0.40±0.06 | 0.06±0.01 | 0.25±0.08 | 0.26±0.08 | 0.47±0.29 |
| Δω (ppm) | -2.84±0.02 | -2.84±0.01 | -2.68±0.05 | -3.04±0.04 | -3.08±0.03 | -3.02±0.02 |
| $k_{ex}$ ($s^{-1}$) | 455±143 | 470±48 | 1151±283 | 993±193 | 864±204 | 354±215 |
| $R_1$ ($s^{-1}$) | 11.71±0.02 | 9.25±0.01 | 8.71±0.01 | 13.09±0.03 | 11.54±0.02 | 11.83±0.02 |
| $R_2$ ($s^{-1}$) | 39.14±0.56 | 27.19±0.09 | 26.34±0.21 | 38.5±0.44 | 32.78±0.27 | 35.07±0.72 |
| Red. $r\chi^2$ | 8.5 | 5.61 | 9.19 | 8.51 | 5.22 | 34.32 |

**Supplementary Table S4.** Comparison between initial guesses and best fit Δω values from a constrained 3-state global fit of the TAR ES2 $R_{1\rho}$ data at pH = 5.4, 6.0, 6.4 and 7.0 assuming a linear topology ($A \rightleftharpoons AH^+ \rightleftharpoons BH^+$). Errors represent the experimental uncertainty in the measured $R_{1\rho}$ data and were computed as ± 1 s.d. from Monte Carlo simulations (number of iterations = 500) for one measurement as previously described.[2]

| pH | Nucleus | Δω | Initial guess | Fit values |
|---|---|---|---|---|
| pH 5.4 | U38-N3 | $\Delta\omega_{A\rightarrow AH+}$ | 0 | -0.4 ± 0.67 |
| | | $\Delta\omega_{A\rightarrow BH+}$ | -5.0 | -4.8 ± 0.1 |
| | U23-C6 | $\Delta\omega_{A\rightarrow AH+}$ | 0 | 0.8 ± 1.0 |
| | | $\Delta\omega_{A\rightarrow BH+}$ | 2.0 | 1.23 ± 2.1 |
| | A35-C8 | $\Delta\omega_{A\rightarrow AH+}$ | 0 | -0.4 ± 1.1 |
| | | $\Delta\omega_{A\rightarrow BH+}$ | -2.3 | -2.5 ± 0.1 |
| pH 6.0 | U38-N3 | $\Delta\omega_{A\rightarrow AH+}$ | 0 | 0.5 ± 0.9 |
| | | $\Delta\omega_{A\rightarrow BH+}$ | -5.0 | -5.5 ± 0.1 |
| | U23-C6 | $\Delta\omega_{A\rightarrow AH+}$ | 0 | 1.2 ± 2.1 |
| | | $\Delta\omega_{A\rightarrow BH+}$ | 2.0 | 2.1 ± 0.1 |
| | A35-C8 | $\Delta\omega_{A\rightarrow AH+}$ | 0 | 0.9 ± 1.0 |
| | | $\Delta\omega_{A\rightarrow BH+}$ | -2.3 | -2.6 ± 0.1 |
| pH 6.4 | U38-N3 | $\Delta\omega_{A\rightarrow AH+}$ | 0 | 0.6 ± 0.9 |
| | | $\Delta\omega_{A\rightarrow BH+}$ | -5.0 | -6.9 ± 0.4 |
| | U23-C6 | $\Delta\omega_{A\rightarrow AH+}$ | 0 | -1.7 ± 2.6 |
| | | $\Delta\omega_{A\rightarrow BH+}$ | 2.0 | 2.4 ± 0.2 |
| | A35-C8 | $\Delta\omega_{A\rightarrow AH+}$ | 0 | -2.0 ± 0.9 |
| | | $\Delta\omega_{A\rightarrow BH+}$ | -2.3 | -2.7 ± 0.1 |
| pH 7.0 | U38-N3 | $\Delta\omega_{A\rightarrow AH+}$ | 0 | 2.8 ± 4.9 |
| | | $\Delta\omega_{A\rightarrow BH+}$ | -5.0 | -5.8 ± 1.7 |
| | U23-C6 | $\Delta\omega_{A\rightarrow AH+}$ | 0 | 2.5 ± 7.7 |
| | | $\Delta\omega_{A\rightarrow BH+}$ | 2.0 | 2.4 ± 0.7 |
| | A35-C8 | $\Delta\omega_{A\rightarrow AH+}$ | 0 | 0.5 ± 2.1 |
| | | $\Delta\omega_{A\rightarrow BH+}$ | -2.3 | -2.4 ± 0.4 |
| pH 7.4 | U38-N3 | $\Delta\omega_{A\rightarrow AH+}$ | 0 | 0.2 ± 1.3 |
| | | $\Delta\omega_{A\rightarrow BH+}$ | -5.0 | -5.4 ± 1.6 |
| | U23-C6 | $\Delta\omega_{A\rightarrow AH+}$ | 0 | -4.5 ± 2.8 |
| | | $\Delta\omega_{A\rightarrow BH+}$ | 2.0 | 2.8 ± 0.8 |
| | A35-C8 | $\Delta\omega_{A\rightarrow AH+}$ | 0 | 0.3 ± 1.6 |
| | | $\Delta\omega_{A\rightarrow BH+}$ | -2.3 | -2.7 ± 1.1 |

**Supplementary Table S5.** List of relaxation delay times ($\tau_{relax}$ in ms), spin-lock powers ($\omega_1/2\pi$, in Hz) and offsets ($\Omega/2\pi$, in Hz) used in off-resonance $^{13}C$ $R_{1\rho}$ experiments.

| Temperature | Delay (ms) | Spectrometer | [$\omega_1/2\pi$ (Hz)] [$\Omega/2\pi$ (Hz)] |
|---|---|---|---|
| **wtTAR (pH 5.4, 25 °C, AUG labeled, 90% $H_2O$:10% $D_2O$)** | | | |
| U38-N3 | 0, 20, 40, 60, 80 | 900 MHz | [200] [-704, -640, -576, -512, -448, -384, -320, -256, -192, -128, -64, -10, 10, 64, 128, 192, 256, 320, 384, 448, 512, 576, 640, 704]<br>[400] [-1397, -1270, -1143, -1016, -889, -762, -635, -508, -381, -254, -127, -10, 10, 127, 254, 381, 508, 635, 762, 889, 1016, 1143, 1270, 1397]<br>[600] [-2101, -1910, -1719, -1528, -1337, -1146, -955, -764, -573, -382, -191, -10, 10, 191, 382, 573, 764, 955, 1146, 1337, 1528, 1719, 1910, 2101]<br>[1000] [-3498, -3180, -2862, -2544, -2226, -1908, -1590, -1272, -954, -636, -318, -10, 10, 318, 636, 954, 1272, 1590, 1908, 2226, 2544, 2862, 3180, 3498]<br>[2000] [-10] |
| U23-C6 | 0, 7, 14, 21, 28 | 900 MHz | [200] [-704, -640, -576, -512, -448, -384, -320, -256, -192, -128, -64, -10, 10, 64, 128, 192, 256, 320, 384, 448, 512, 576, 640, 704]<br>[400] [-1397, -1270, -1143, -1016, -889, -762, -635, -508, -381, -254, -127, -10, 10, 127, 254, 381, 508, 635, 762, 889, 1016, 1143, 1270, 1397]<br>[600] [-2101, -1910, -1719, -1528, -1337, -1146, -955, -764, -573, -382, -191, -10, 10, 191, 382, 573, 764, 955, 1146, 1337, 1528, 1719, 1910, 2101]<br>[1000] [-3498, -3180, -2862, -2544, -2226, -1908, -1590, -1272, -954, -636, -318, -10, 10, 318, 636, 954, 1272, 1590, 1908, 2226, 2544, 2862, 3180, 3498] |
| A35-C8 | 0, 10, 20, 30, 40 | 900 MHz | [200] [-704, -640, -576, -512, -448, -384, -320, -256, -192, -128, -64, -10, 10, 64, 128, 192, 256, 320, 384, 448, 512, 576, 640, 704]<br>[400] [-1397, -1270, -1143, -1016, -889, -762, -635, -508, -381, -254, -127, -10, 10, 127, 254, 381, 508, 635, 762, 889, 1016, 1143, |

| | | | |
|---|---|---|---|
| | | | 1270, 1397]<br>[600] [-2101, -1910, -1719, -1528, -1337, -1146, -955, -764, -573, -382, -191, -10, 10, 191, 382, 573, 764, 955, 1146, 1337, 1528, 1719, 1910, 2101]<br>[1200] [-4202, -3820, -3438, -3056, -2674, -2292, -1910, -1528, -1146, -764, -382, -10, 10, 382, 764, 1146] |
| **wtTAR (pH 5.4, 25 °C, C labeled, 90% $H_2O$:10% $D_2O$)** | | | |
| C24-C5 | 0, 5, 10, 15, 20, 25, 30, 35, 40 | 600 MHz | [150] [-350, -300, -250, -200, -150, -125, -100, -50, -10, 0, 10, 50, 100, 125, 150, 200, 250, 300, 350]<br>[250] [-500, -400, -300, -250, -200, -150, -100, -50, 0, 50, 100, 150, 200, 250, 300, 400, 500]<br>[400] [-1000, -800, -600, -500, -400, -300, -200, -100, -50, 0, 50, 100, 200, 300, 400, 500, 600, 800, 1000]<br>[600] [-1600, -1400, -1200, -1000, -800, -600, -400, -200, -100, -50, 0, 50, 100, 200, 400, 600, 800, 1000, 1200, 1400, 1600] |
| C24-C6 | 0, 5, 10, 15, 20, 25, 30, 35, 40 | 600 MHz | |
| C24-C1′ | 0, 4, 8, 13, 17, 21, 26, 30, 35 | 600 MHz | |
| **wtTAR (pH 5.4, 25 °C, fully labeled, 90% $H_2O$:10% $D_2O$)** | | | |
| C39-C5 | 0, 10, 20, 40 | 800 MHz | [150] [-528, -480, -432, -384, -336, -288, -240, -192, -144, -96, -48, -10, 10, 48, 96, 144, 192, 240, 288, 336, 384, 432, 480, 528]<br>[250] [-880, -800, -720, -640, -560, -480, -400, -320, -240, -160, -80, -10, 10, 80, 160, 240, 320, 400, 480, 560, 640, 720, 800, 880]<br>[400] [-1397, -1270, -1143, -1016, -889, -762, -635, -508, -381, -254, -127, -10, 10, 127, 254, 381, 508, 635, 762, 889, 1016, 1143, 1270, 1397]<br>[600] [-2101, -1910, -1719, -1528, -1337, -1146, -955, -764, -573, -382, -191, -10, 10, 191, 382, 573, 764, 955, 1146, 1337, 1528, 1719, 1910, 2101] |
| **wtTAR (pH 6.0, 25 °C, fully labeled, 90% $H_2O$:10% $D_2O$)** | | | |
| U38-N3 | 0, 30, 60, 90, 120 | 900 MHz | [200] [-704, -640, -576, -512, -448, -384, -320, -256, -192, -128, -64, -10, 10, 64, 128, 192, 256, 320, 384, 448, 512, 576, 640, 704] |

| | | | |
|---|---|---|---|
| | | | [400] [-1397, -1270, -1143, -1016, -889, -762, -635, -508, -381, -254, -127, -10, 10, 127, 254, 381, 508, 635, 762, 889, 1016, 1143, 1270, 1397]<br>[600] [-2101, -1910, -1719, -1528, -1337, -1146, -955, -764, -573, -382, -191, -10, 10, 191, 382, 573, 764, 955, 1146, 1337, 1528, 1719, 1910, 2101]<br>[1000] [-3498, -3180, -2862, -2544, -2226, -1908, -1590, -1272, -954, -636, -318, -10, 10, 318, 636, 954, 1272, 1590, 1908, 2226, 2544, 2862, 3180, 3498] |
| U23-C6 | 0, 11, 22, 33, 44 | 900 MHz | [200] [-704, -640, -576, -512, -448, -384, -320, -256, -192, -128, -64, -10, 10, 64, 128, 192, 256, 320, 384, 448, 512, 576, 640, 704]<br>[400] [-1397, -1270, -1143, -1016, -889, -762, -635, -508, -381, -254, -127, -10, 10, 127, 254, 381, 508, 635, 762, 889, 1016, 1143, 1270, 1397]<br>[600] [-2101, -1910, -1719, -1528, -1337, -1146, -955, -764, -573, -382, -191, -10, 10, 191, 382, 573, 764, 955, 1146, 1337, 1528, 1719, 1910, 2101]<br>[1000] [-3498, -3180, -2862, -2544, -2226, -1908, -1590, -1272, -954, -636, -318, -10, 10, 318, 636, 954, 1272, 1590, 1908, 2226, 2544, 2862, 3180, 3498] |
| A35-C8 | 0, 15, 30, 45, 60 | 900 MHz | [150] [-528, -480, -432, -384, -336, -288, -240, -192, -144, -96, -48, -10, 10, 48, 96, 144, 192, 240, 288, 336, 384, 432, 480, 528]<br>[200] [-704, -640, -576, -512, -448, -384, -320, -256, -192, -128, -64, -10, 10, 64, 128, 192, 256, 320, 384, 448, 512, 576, 640, 704]<br>[300] [-1045, -950, -855, -760, -665, -570, -475, -380, -285, -190, -95, -10, 10, 95, 190, 285, 380, 475, 570, 665, 760, 855, 950, 1045]<br>[400] [-1397, -1270, -1143, -1016, -889, -762, -635, -508, -381, -254, -127, -10, 10, 127, 254, 381, 508, 635, 762, 889, 1016, 1143, 1270, 1397]<br>[600] [-2101, -1910, -1719, -1528, -1337, - |

| | | | |
|---|---|---|---|
| | | | 1146, -955, -764, -573, -382, -191, -10, 10, 191, 382, 573, 764, 955, 1146, 1337, 1528, 1719, 1910, 2101]<br>[1200] [-4202, -3820, -3438, -3056, -2674, -2292, -1910, -1528, -1146, -764, -382, -10, 10, 382, 764, 1146, 1528, 1910, 2292, 2674, 3056, 3438, 3820, 4202] |
| **wtTAR (pH 6.4, 25 °C, fully labeled, 90% $H_2O$:10% $D_2O$)** | | | |
| U38-N3 | 0, 30, 60, 90, 120 | 600 MHz | [200] [-704, -640, -576, -512, -448, -384, -320, -256, -192, -128, -64, -10, 10, 64, 128, 192, 256, 320, 384, 448, 512, 576, 640, 704]<br>[400] [-1397, -1270, -1143, -1016, -889, -762, -635, -508, -381, -254, -127, -10, 10, 127, 254, 381, 508, 635, 762, 889, 1016, 1143, 1270, 1397]<br>[800] [-2805, -2550, -2295, -2040, -1785, -1530, -1275, -1020, -765, -510, -255, -10, 10, 255, 510, 765, 1020, 1275, 1530, 1785, 2040, 2295, 2550, 2805]<br>[1200] [-4202, -3820, -3438, -3056, -2674, -2292, -1910, -1528, -1146, -764, -382, -10, 10, 382, 764, 1146, 1528, 1910, 2292, 2674, 3056, 3438, 3820, 4202] |
| U23-C6 | 0, 11, 22, 33, 44 | 600 MHz | [200] [-704, -640, -576, -512, -448, -384, -320, -256, -192, -128, -64, -10, 10, 64, 128, 192, 256, 320, 384, 448, 512, 576, 640, 704]<br>[400] [-1397, -1270, -1143, -1016, -889, -762, -635, -508, -381, -254, -127, -10, 10, 127, 254, 381, 508, 635, 762, 889, 1016, 1143, 1270, 1397]<br>[600] [-2101, -1910, -1719, -1528, -1337, -1146, -955, -764, -573, -382, -191, -10, 10, 191, 382, 573, 764, 955, 1146, 1337, 1528, 1719, 1910, 2101]<br>[1000] [-3498, -3180, -2862, -2544, -2226, -1908, -1590, -1272, -954, -636, -318, -10, 10, 318, 636, 954, 1272, 1590, 1908, 2226, 2544, 2862, 3180, 3498] |
| A35-C8 | 0, 15, 30, 45, 60 | 600 MHz | [150] [-528, -480, -432, -384, -336, -288, -240, -192, -144, -96, -48, -10, 10, 48, 96, 144, 192, 240, 288, 336, 384, 432, 480, 528] |

| | | | |
|---|---|---|---|
| | | | [200] [-704, -640, -576, -512, -448, -384, -320, -256, -192, -128, -64, -10, 10, 64, 128, 192, 256, 320, 384, 448, 512, 576, 640, 704]<br>[300] [-1045, -950, -855, -760, -665, -570, -475, -380, -285, -190, -95, -10, 10, 95, 190, 285, 380, 475, 570, 665, 760, 855, 950, 1045]<br>[400] [-1397, -1270, -1143, -1016, -889, -762, -635, -508, -381, -254, -127, -10, 10, 127, 254, 381, 508, 635, 762, 889, 1016, 1143, 1270, 1397]<br>[800] [-2805, -2550, -2295, -2040, -1785, -1530, -1275, -1020, -765, -510, -255, -10, 10, 255, 510, 765, 1020, 1275, 1530, 1785, 2040, 2295, 2550, 2805] |
| **wtTAR (pH 7.0, 25 °C, fully labeled, 90% $H_2O$:10% $D_2O$)** | | | |
| U38-N3 | 0, 30, 60, 90, 120 | 800 MHz | [200] [-704, -640, -576, -512, -448, -384, -320, -256, -192, -128, -64, -10, 10, 64, 128, 192, 256, 320, 384, 448, 512, 576, 640, 704]<br>[400] [-1397, -1270, -1143, -1016, -889, -762, -635, -508, -381, -254, -127, -10, 10, 127, 254, 381, 508, 635, 762, 889, 1016, 1143, 1270, 1397]<br>[600] [-2101, -1910, -1719, -1528, -1337, -1146, -955, -764, -573, -382, -191, -10, 10, 191, 382, 573, 764, 955, 1146, 1337, 1528, 1719, 1910, 2101]<br>[800] [-2805, -2550, -2295, -2040, -1785, -1530, -1275, -1020, -765, -510, -255, -10, 10, 255, 510, 765, 1020, 1275, 1530, 1785, 2040, 2295, 2550, 2805]<br>[1000] [-3498, -3180, -2862, -2544, -2226, -1908, -1590, -1272, -954, -636, -318, -10, 10, 318, 636, 954, 1272, 1590, 1908, 2226, 2544, 2862, 3180, 3498] |
| U23-C6 | 0, 11, 22, 33, 44 | 800 MHz | [200] [-704, -640, -576, -512, -448, -384, -320, -256, -192, -128, -64, -10, 10, 64, 128, 192, 256, 320, 384, 448, 512, 576, 640, 704]<br>[400] [-1397, -1270, -1143, -1016, -889, -762, -635, -508, -381, -254, -127, -10, 10, 127, 254, 381, 508, 635, 762, 889, 1016, 1143, 1270, 1397] |

| | | | |
|---|---|---|---|
| | | | [600] [-2101, -1910, -1719, -1528, -1337, -1146, -955, -764, -573, -382, -191, -10, 10, 191, 382, 573, 764, 955, 1146, 1337, 1528, 1719, 1910, 2101]<br>[800] [-2805, -2550, -2295, -2040, -1785, -1530, -1275, -1020, -765, -510, -255, -10, 10, 255, 510, 765, 1020, 1275, 1530, 1785, 2040, 2295, 2550, 2805]<br>[1000] [-3498, -3180, -2862, -2544, -2226, -1908, -1590, -1272, -954, -636, -318, -10, 10, 318, 636, 954, 1272, 1590, 1908, 2226, 2544, 2862, 3180, 3498] |
| A35-C8 | 0, 15, 30, 45, 60 | 800 MHz | [150] [-528, -480, -432, -384, -336, -288, -240, -192, -144, -96, -48, -10, 10, 48, 96, 144, 192, 240, 288, 336, 384, 432, 480, 528]<br>[200] [-704, -640, -576, -512, -448, -384, -320, -256, -192, -128, -64, -10, 10, 64, 128, 192, 256, 320, 384, 448, 512, 576, 640, 704]<br>[400] [-1397, -1270, -1143, -1016, -889, -762, -635, -508, -381, -254, -127, -10, 10, 127, 254, 381, 508, 635, 762, 889, 1016, 1143, 1270, 1397]<br>[600] [-2101, -1910, -1719, -1528, -1337, -1146, -955, -764, -573, -382, -191, -10, 10, 191, 382, 573, 764, 955, 1146, 1337, 1528, 1719, 1910, 2101]<br>[800] [-2805, -2550, -2295, -2040, -1785, -1530, -1275, -1020, -765, -510, -255, -10, 10, 255, 510, 765, 1020, 1275, 1530, 1785, 2040, 2295, 2550, 2805]<br>[1000] [-3498, -3180, -2862, -2544, -2226, -1908, -1590, -1272, -954, -636, -318, -10, 10, 318, 636, 954, 1272, 1590, 1908, 2226, 2544, 2862, 3180, 3498] |
| **wtTAR (pH 7.4, 25 °C, AUG labeled, 90% $H_2O$:10% $D_2O$)** | | | |
| U38-N3 | 0, 30, 60, 90, 120 | 900 MHz | [200] [-704, -640, -576, -512, -448, -384, -320, -256, -192, -128, -64, -10, 10, 64, 128, 192, 256, 320, 384, 448, 512, 576, 640, 704]<br>[400] [-1397, -1270, -1143, -1016, -889, -762, -635, -508, -381, -254, -127, -10, 10, 127, 254, 381, 508, 635, 762, 889, 1016, 1143, 1270, 1397] |

| | | | |
|---|---|---|---|
| | | | [600] [-2101, -1910, -1719, -1528, -1337, -1146, -955, -764, -573, -382, -191, -10, 10, 191, 382, 573, 764, 955, 1146, 1337, 1528, 1719, 1910, 2101]<br>[1000] [-3498, -3180, -2862, -2544, -2226, -1908, -1590, -1272, -954, -636, -318, -10, 10, 318, 636, 954, 1272, 1590, 1908, 2226, 2544, 2862, 3180, 3498]<br>[2000] [-636, -10] |
| U23-C6 | 0, 10, 20, 30, 40 | 900 MHz | [200] [-704, -640, -576, -512, -448, -384, -320, -256, -192, -128, -64, -10, 10, 64, 128, 192, 256, 320, 384, 448, 512, 576, 640, 704]<br>[400] [-1397, -1270, -1143, -1016, -889, -762, -635, -508, -381, -254, -127, -10, 10, 127, 254, 381, 508, 635, 762, 889, 1016, 1143, 1270, 1397]<br>[600] [-2101, -1910, -1719, -1528, -1337, -1146, -955, -764, -573, -382, -191, -10, 10, 191, 382, 573, 764, 955, 1146, 1337, 1528, 1719, 1910, 2101]<br>[1000] [-3498, -3180, -2862, -2544, -2226, -1908, -1590, -1272, -954, -636, -318, -10, 10, 318, 636, 954, 1272, 1590, 1908, 2226, 2544, 2862, 3180, 3498] |
| A35-C8 | 0, 10, 20, 30, 40, 50 | 900 MHz | [200] [-704, -640, -576, -512, -448, -384, -320, -256, -192, -128, -64, -10, 10, 64, 128, 192, 256, 320, 384, 448, 512, 576, 640, 704]<br>[400] [-1397, -1270, -1143, -1016, -889, -762, -635, -508, -381, -254, -127, -10, 10, 127, 254, 381, 508, 635, 762, 889, 1016, 1143, 1270, 1397]<br>[600] [-2101, -1910, -1719, -1528, -1337, -1146, -955, -764, -573, -382, -191, -10, 10, 191, 382, 573, 764, 955, 1146, 1337, 1528, 1719, 1910, 2101]<br>[800] [-2805, -2550, -2295, -2040, -1785, -1530, -1275, -1020, -765, -510, -255, -10, 10, 255, 510, 765, 1020, 1275, 1530, 1785, 2040, 2295, 2550, 2805]<br>[1200] [-4202, -3820, -3438, -3056, -2674, -2292, -1910, -1528, -1146, -764, -382, -10, 10, 382, 764, 1146, 1528, 1910, 2292, 2674, |

| | | | |
|---|---|---|---|
| | | | 3056, 3438, 3820, 4202]<br>[2000] [-6996, -6360, -5724, -5088, -4452, -3816, -3180, -2544, -1908, -1272, -636, -10, 10, 636, 1272, 1908, 2544, 3180, 3816, 4452, 5088, 5724, 6360, 6996] |
| **wtTAR (pH 8.4, 25 °C, AUG labeled, 90% $H_2O$:10% $D_2O$)** | | | |
| U38-N3 | 0, 30, 60, 90, 120 | 900 MHz | [100] [-352, -320, -288, -256, -224, -192, -160, -128, -96, -64, -32, -10, 10, 32, 64, 96, 128, 160, 192, 224, 256, 288, 320, 352]<br>[200] [-704, -640, -576, -512, -448, -384, -320, -256, -192, -128, -64, -10, 10, 64, 128, 192, 256, 320, 384, 448, 512, 576, 640, 704]<br>[400] [-1397, -1270, -1143, -1016, -889, -762, -635, -508, -381, -254, -127, -10, 10, 127, 254, 381, 508, 635, 762, 889, 1016, 1143, 1270, 1397]<br>[600] [-2101, -1910, -1719, -1528, -1337, -1146, -955, -764, -573, -382, -191, -10, 10, 191, 382, 573, 764, 955, 1146, 1337, 1528, 1719, 1910, 2101]<br>[1000] [-3498, -3180, -2862, -2544, -2226, -1908, -1590, -1272, -954, -636, -318, -10, 10, 318, 636, 954, 1272, 1590, 1908, 2226, 2544, 2862, 3180, 3498]<br>[2000] [-6996, -6360, -5724, -5088, -4452, -3816, -3180, -2544, -1908, -1272, -636, -10, 10, 636, 1272, 1908, 2544, 3180, 3816, 4452, 5088, 5724, 6360, 6996] |
| U23-C6 | 0, 12, 24, 36 | 900 MHz | [200] [-704, -640, -576, -512, -448, -384, -320, -256, -192, -128, -64, -10, 10, 64, 128, 192, 256, 320, 384, 448, 512, 576, 640, 704]<br>[400] [-1397, -1270, -1143, -1016, -889, -762, -635, -508, -381, -254, -127, -10, 10, 127, 254, 381, 508, 635, 762, 889, 1016, 1143, 1270, 1397]<br>[600] [-2101, -1910, -1719, -1528, -1337, -1146, -955, -764, -573, -382, -191, -10, 10, 191, 382, 573, 764, 955, 1146, 1337, 1528, 1719, 1910, 2101]<br>[1000] [-3498, -3180, -2862, -2544, -2226, -1908, -1590, -1272, -954, -636, -318, -10, 10, |

| | | | |
|---|---|---|---|
| | | | 318, 636, 954, 1272, 1590, 1908, 2226, 2544, 2862, 3180, 3498] |

**Supplementary Table S6.** List of relaxation delay times ($\tau_{relax}$ in ms), spin-lock powers ($\omega_1/2\pi$, in Hz) and offsets ($\Omega/2\pi$, in Hz) used in off-resonance $^{15}N$ and $^{13}C$ $R_{1\rho}$ experiments measured on the $TAR^{ES2}$ construct.

| Temperature | Delay (ms) | Spectrometer | [$\omega_1/2\pi$ (Hz)] [$\Omega/2\pi$ (Hz)] |
|---|---|---|---|
| **$TAR^{ES2}$ (pH 5.4, 25 °C, AUG labeled, 90% $H_2O$:10% $D_2O$)** | | | |
| U38-N3 | 0, 25, 50, 75, 100 | 900 MHz | [200] [-704, -640, -576, -512, -448, -384, -320, -256, -192, -128, -64, -10, 10, 64, 128, 192, 256, 320, 384, 448, 512, 576, 640, 704]<br>[400] [-1397, -1270, -1143, -1016, -889, -762, -635, -508, -381, -254, -127, -10, 10, 127, 254, 381, 508, 635, 762, 889, 1016, 1143, 1270, 1397]<br>[600] [-2101, -1910, -1719, -1528, -1337, -1146, -955, -764, -573, -382, -191, -10, 10, 191, 382, 573, 764, 955, 1146, 1337, 1528, 1719, 1910, 2101]<br>[1000] [-3498, -3180, -2862, -2544, -2226, -1908, -1590, -1272, -954, -636, -318, -10, 10, 318, 636, 954, 1272, 1590, 1908, 2226, 2544, 2862, 3180, 3498] |
| U23-C6 | 0, 10, 20, 30, 40 | 900 MHz | [200] [-704, -640, -576, -512, -448, -384, -320, -256, -192, -128, -64, -10, 10, 64, 128, 192, 256, 320, 384, 448, 512, 576, 640, 704]<br>[400] [-1397, -1270, -1143, -1016, -889, -762, -635, -508, -381, -254, -127, -10, 10, 127, 254, 381, 508, 635, 762, 889, 1016, 1143, 1270, 1397]<br>[600] [-2101, -1910, -1719, -1528, -1337, -1146, -955, -764, -573, -382, -191, -10, 10, 191, 382, 573, 764, 955, 1146, 1337, 1528, 1719, 1910, 2101]<br>[1200] [-4202, -3820, -3438, -3056, -2674, -2292, -1910, -1528, -1146, -764, -382, -10, 10, 382, 764, 1146, 1528, 1910, 2292, 2674, 3056, 3438, 3820, 4202] |
| **$TAR^{ES2}$ (pH 6.0, 25 °C, fully labeled, 90% $H_2O$:10% $D_2O$)** | | | |

| | | | |
|---|---|---|---|
| U38-N3 | 0, 15, 30, 45, 60 | 900 MHz | [200] [-704, -640, -576, -512, -448, -384, -320, -256, -192, -128, -64, -10, 10, 64, 128, 192, 256, 320, 384, 448, 512, 576, 640, 704]<br>[400] [-1397, -1270, -1143, -1016, -889, -762, -635, -508, -381, -254, -127, -10, 10, 127, 254, 381, 508, 635, 762, 889, 1016, 1143, 1270, 1397]<br>[600] [-2101, -1910, -1719, -1528, -1337, -1146, -955, -764, -573, -382, -191, -10, 10, 191, 382, 573, 764, 955, 1146, 1337, 1528, 1719, 1910, 2101]<br>[1000] [-3498, -3180, -2862, -2544, -2226, -1908, -1590, -1272, -954, -636, -318, -10, 10, 318, 636, 954, 1272, 1590, 1908, 2226, 2544, 2862, 3180, 3498] |
| U23-C6 | 0, 8, 16, 24, 32 | 900 MHz | [200] [-704, -640, -576, -512, -448, -384, -320, -256, -192, -128, -64, -10, 10, 64, 128, 192, 256, 320, 384, 448, 512, 576, 640, 704]<br>[400] [-1397, -1270, -1143, -1016, -889, -762, -635, -508, -381, -254, -127, -10, 10, 127, 254, 381, 508, 635, 762, 889, 1016, 1143, 1270, 1397]<br>[600] [-2101, -1910, -1719, -1528, -1337, -1146, -955, -764, -573, -382, -191, -10, 10, 191, 382, 573, 764, 955, 1146, 1337, 1528, 1719, 1910, 2101]<br>[1200] [-4202, -3820, -3438, -3056, -2674, -2292, -1910, -1528, -1146, -764, -382, -10, 10, 382, 764, 1146, 1528, 1910, 2292, 2674, 3056, 3438, 3820, 4202] |
| **$TAR^{ES2}$ (pH 6.4, 25 °C, fully labeled, 90% $H_2O$:10% $D_2O$)** | | | |
| U38-N3 | 0, 20, 40, 60 | 900 MHz | [200] [-704, -640, -576, -512, -448, -384, -320, -256, -192, -128, -64, -10, 10, 64, 128, 192, 256, 320, 384, 448, 512, 576, 640, 704]<br>[400] [-1397, -1270, -1143, -1016, -889, -762, -635, -508, -381, -254, -127, -10, 10, 127, 254, 381, 508, 635, 762, 889, 1016, 1143, 1270, 1397]<br>[600] [-2101, -1910, -1719, -1528, -1337, -1146, -955, -764, -573, -382, -191, -10, 10, 191, 382, 573, 764, 955, 1146, 1337, 1528, 1719, 1910, 2101] |

| | | | |
|---|---|---|---|
| | | | [1000] [-3498, -3180, -2862, -2544, -2226, -1908, -1590, -1272, -954, -636, -318, -10, 10, 318, 636, 954, 1272, 1590, 1908, 2226, 2544, 2862, 3180, 3498] |
| U23-C6 | 0, 5, 10, 15, 20 | 900 MHz | [200] [-704, -640, -576, -512, -448, -384, -320, -256, -192, -128, -64, -10, 10, 64, 128, 192, 256, 320, 384, 448, 512, 576, 640, 704]<br>[400] [-1397, -1270, -1143, -1016, -889, -762, -635, -508, -381, -254, -127, -10, 10, 127, 254, 381, 508, 635, 762, 889, 1016, 1143, 1270, 1397]<br>[600] [-2101, -1910, -1719, -1528, -1337, -1146, -955, -764, -573, -382, -191, -10, 10, 191, 382, 573, 764, 955, 1146, 1337, 1528, 1719, 1910, 2101]<br>[1200] [-4202, -3820, -3438, -3056, -2674, -2292, -1910, -1528, -1146, -764, -382, -10, 10, 382, 764, 1146, 1528, 1910, 2292, 2674, 3056, 3438, 3820, 4202] |

**Supplementary Table S7.** List of relaxation delay times ($\tau_{relax}$ in ms), spin-lock powers ($\omega_1/2\pi$, in Hz) and offsets ($\Omega/2\pi$, in Hz) used in $^1$H CEST experiments.

| T (°C) | $\omega_1$ $2\pi^{-1}$ ($s^{-1}$) | $\Omega$ $2\pi^{-1}$($s^{-1}$) |
|---|---|---|
| **$^1$H CEST wtTAR (pH 5.4, 90% $H_2O$:10% $D_2O$)** | | |
| 25 °C, $\tau_{relax}$ = 100 ms, 700MHz | 10 | [-4137, -3837, -3536, -3236, -3166, -3096, -3026, -2956, -2886, -2816, -2746, -2676, -2606, -2536, -2466, -2396, -2326, -2256, -2186, -2116, -2046, -1976, -1906, -1836, -1766, -1696, -1626, -1556, -1486, -1416, -1346, -1276, -1206, -1136, -1075, -1014, -953, -892, -831, -770, -709, -648, -588, -527, -466, -405, -344, -283, -222, -161, -100, -39, 21, 82, 143, 204, 265, 335, 405, 475, 545, 615, 685, 755, 825, 895, 965, 1035, 1105, 1175, 1245, 1315, 1385, 1455, 1525, 1596, 1666, 1736, 1806, 1876, 1946, 2016, 2086, 2156, 2226, 2296, 2366, 2666, 2966, 3266, 3566, 3866, 4167] |
| | 50 | |
| | 100 | |
| | 200 | |
| | 500 | |
| | 1000 | |
| 25 °C, $\tau_{relax}$ = 100 ms, 700MHz | 10 | [-4145, -3845, -3545, -3245, -3175, -3105, -3035, -2965, -2895, -2825, -2755, -2685, -2615, -2545, -2475, -2405, -2335, -2265, -2195, -2125, -2055, -1985, -1915, -1845, -1775, -1705, -1635, -1564, -1494, -1424, -1354, -1284, -1214, -1144, -1083, -1023, -962, -901, -840, -779, -718, -657, -596, -535, -475, -414, -353, -292, -231, -170, -109, -48, 13, 74, 134, 195, 256, 326, 396, 466, 536, 606, 676, 746, 816, 886, 956, 1027, 1097, 1167, 1237, 1307, 1377, 1447, 1517, 1587, 1657, 1727, 1797, 1867, 1937, 2007, 2077, 2147, 2217, 2287, 2357, 2657, 2957, 3257, 3557, 3858, 4158] |
| | 50 | |
| | 100 | |
| | 200 | |
| | 500 | |
| | 1000 | |
| | 4000 | |
| **$^1$H CEST C24G (pH 5.4, 90% $H_2O$:10% $D_2O$)** | | |
| 25 °C, $\tau_{relax}$ = 100 ms, 700MHz | 10 | [-4190, -3890, -3590, -3289, -3219, -3149, -3079, -3009, -2939, -2869, -2799, -2729, -2659, -2589, -2519, -2449, -2379, -2309, -2239, -2169, -2099, -2029, -1959, -1889, -1819, -1749, -1679, -1609, -1539, -1469, -1399, -1329, -1259, -1189, -1128, -1067, -1006, -945, -884, -823, -762, -701, -641, -580, -519, -458, -397, -336, -275, -214, -153, -93, -32, 29, 90, 151, 212, 282, 352, 422, 492, 562, 632, 702, 772, 842, 912, 982, 1052, 1122, 1192, 1262, 1332, 1402, 1472, 1542, 1612, 1682, 1753, 1823, 1893, 1963, 2033, 2103, 2173, 2243, 2313, 2613, 2913, 3213, 3513, 3813, 4113] |
| | 50 | |
| | 200 | |
| | 500 | |
| | 1000 | |
| **$^1$H CEST C24G (pH 6.4, 90% $H_2O$:10% $D_2O$)** | | |
| | 10 | [-3592, -3335, -3078, -2821, -2761, -2701, -2641, -2581, -2521, -2461, -2401, -2341, -2281, -2221, -2161, -2101, - |

| | | |
|---|---|---|
| 25 °C, $\tau_{relax}$ = 100 ms, 600MHz | 50 | 2041, -1981, -1921, -1861, -1802, -1742, -1682, -1622, -1562, -1502, -1442, -1382, -1322, -1262, -1202, -1142, -1082, -1022, -962, -902, -842, -782, -722, -662, -602, -542, -482, -422, -362, -302, -242, -182, -122, -62, -3, 57, 117, 177, 237, 297, 357, 417, 477, 537, 597, 657, 717, 777, 837, 897, 957, 1017, 1077, 1137, 1197, 1257, 1317, 1377, 1437, 1497, 1557, 1617, 1677, 1736, 1796, 1856, 1916, 1976, 2233, 2490, 2747, 3004, 3261, 3518] |
| | 200 | |
| | 500 | |
| **$^1$H CEST C24G (pH 7.4, 90% $H_2O$:10% $D_2O$)** | | |
| 25 °C, $\tau_{relax}$ = 100 ms, 700MHz | 10 | [-4193, -3893, -3592, -3292, -3222, -3152, -3082, -3012, -2942, -2872, -2802, -2732, -2662, -2592, -2522, -2452, -2382, -2312, -2242, -2172, -2102, -2032, -1962, -1892, -1822, -1752, -1682, -1612, -1542, -1472, -1402, -1332, -1261, -1191, -1131, -1070, -1009, -948, -887, -826, -765, -704, -643, -583, -522, -461, -400, -339, -278, -217, -156, -95, -34, 26, 87, 148, 209, 279, 349, 419, 489, 559, 629, 699, 769, 839, 909, 979, 1049, 1119, 1189, 1259, 1330, 1400, 1470, 1540, 1610, 1680, 1750, 1820, 1890, 1960, 2030, 2100, 2170, 2240, 2310, 2610, 2910, 3210, 3510, 3810, 4111] |
| | 50 | |
| | 100 | |
| | 200 | |
| | 500 | |
| | 1000 | |

## References


1 Geng, A. *et al.* An RNA excited conformational state at atomic resolution. *Nat. Commun.* **14**, 8432 (2023). https://doi.org/10.1038/s41467-023-43673-6

2 Rangadurai, A., Szymaski, E. S., Kimsey, I. J., Shi, H. & Al-Hashimi, H. M. Characterizing micro-to-millisecond chemical exchange in nucleic acids using off-resonance R(1rho) relaxation dispersion. *Prog Nucl Magn Reson Spectrosc* **112-113**, 55-102 (2019). https://doi.org/10.1016/j.pnmrs.2019.05.002
